# Supplementary material for: Increased neutralization and IgG epitope identification after MVA-MERS-S booster vaccination against Middle East respiratory syndrome
Source: Nat Commun. 2022 Jul 19;13:4182. doi: 10.1038/s41467-022-31557-0 (PMC9295877; doi:10.1038/s41467-022-31557-0)
Supplement: Supplementary file 1 — Supplementary Information [file 41467_2022_31557_MOESM1_ESM.pdf]

Supplementary Appendix

Increased neutralization and IgG epitope identification after MVA-MERS-S booster vaccination against Middle East respiratory syndrome

Anahita Fathi,[1,2,3,4] Christine Dahlke,[1,2,3] Verena Krähling,[5,6] Alexandra Kupke,[5,6] Nisreen M. A. Okba,[7] Matthijs P. Raadsen,[7] Jasmin Heidepriem,[8] Marcel A. Müller,[9,10] Grigori Paris,[8] Susan Lassen,[1,2,3] Michael Klüver,[5,6] Asisa Volz,[11,12] Till Koch,[2,3,4] My L. Ly,[1,2,3] Monika Friedrich,[1,2,3] Robert Fux,[13,14] Alina Tscherne,[13,14] Georgia Kalodimou,[13,14] Stefan Schmiedel,[3,4] Victor M. Corman,[9,10] Thomas Hesterkamp,[15] Christian Drosten,[9,10] Felix F. Loeffler,[8] Bart L. Haagmans,[7] Gerd Sutter,[13,14] Stephan Becker,[5,6] Marylyn M. Addo[1,2,3]*

[1]University Medical Center Hamburg-Eppendorf, Institute for Infection Research and Vaccine Development (IIRVD); Hamburg, Germany.

[2]Bernhard-Nocht-Institute for Tropical Medicine, Department for Clinical Immunology of Infectious Diseases; Hamburg, Germany.

[3]German Center for Infection Research, partner site Hamburg-Lübeck-Borstel-Riems; Hamburg, Germany.

[4]University Medical Center Hamburg-Eppendorf, First Department of Medicine, Division of Infectious Diseases; Hamburg, Germany.

[5]Philipps University Marburg, Institute of Virology; Marburg, Germany.

[6]German Center for Infection Research, partner site Gießen-Marburg-Langen; Marburg, Germany.

[7]Erasmus Medical Center, Department of Viroscience; Rotterdam, the Netherlands.

[8]Max Planck Institute of Colloids and Interfaces, Department of Biomolecular Systems; Potsdam, Germany.

[9]Charité-Universitätsmedizin Berlin, corporate member of Freie Universität Berlin, Humboldt-Universität zu Berlin, and Berlin Institute of Health, Institute of Virology; Berlin, Germany.

[10]German Center for Infection Research, partner site Berlin; Berlin, Germany.

[11]University of Veterinary Medicine Hanover, Institute of Virology; Hanover, Germany.

[12]German Center for Infection Research, partner site Hanover-Brunswick; Hanover, Germany.

[13]LMU University of Munich, Institute of Infectious Diseases and Zoonoses; Munich, Germany.

[14]German Center for Infection Research, partner site Munich; Munich, Germany.

[15]German Center for Infection Research, Translational Project Management Office; Brunswick, Germany.

Contents

1 FIGURES 3
1.1 Supplementary Fig. 1. Comparison of AE after each vaccination (former high dose cohort). 3
1.2 Supplementary Fig. 2. Relative changes of hematologic parameters and CRP after vaccination. 4
1.3 Supplementary Fig. 3. Correlation of neutralizing anti-MERS-CoV antibody levels measured by VNT. 5
1.4 Supplementary Fig. 4. MERS-CoV-S peptide microarray including unvaccinated control. 6
1.5 Supplementary Fig. 5. Longitudinal development of immunogenic anti-MERS-S IgG responses (RBD). 7
1.6 Supplementary Fig. 6. Longitudinal development of immunogenic anti-MERS-S IgG responses (stem helix). 8
1.7 Supplementary Fig. 7. Correlation of anti-MERS-S antibody titers measured by IFA to neutralizing antibody levels. 9
2 TABLES 10
2.1 Supplementary Table 1. Baseline characteristics of study participants. 10
2.2 Supplementary Table 2. Frequency and severity of adverse events after late booster immunization. 11
2.3 Supplementary Table 3. Percentage of participants with positive immunofluorescence titers at baseline (Day 0). 12
2.4 Supplementary Table 4. Relationship between anti-HCoV-S baseline titers (D0) and anti-MERS-CoV-S titers on Day 42 in IFA. 12
2.5 Supplementary Table 5. Relationship between anti-HCoV-S and anti-MERS-CoV-S titer fold-change on Day 42 in IFA. 12
3. Study Protocol 12

1 FIGURES

1.1 Supplementary Fig. 1. Comparison of AEs after each vaccination (former high dose cohort).

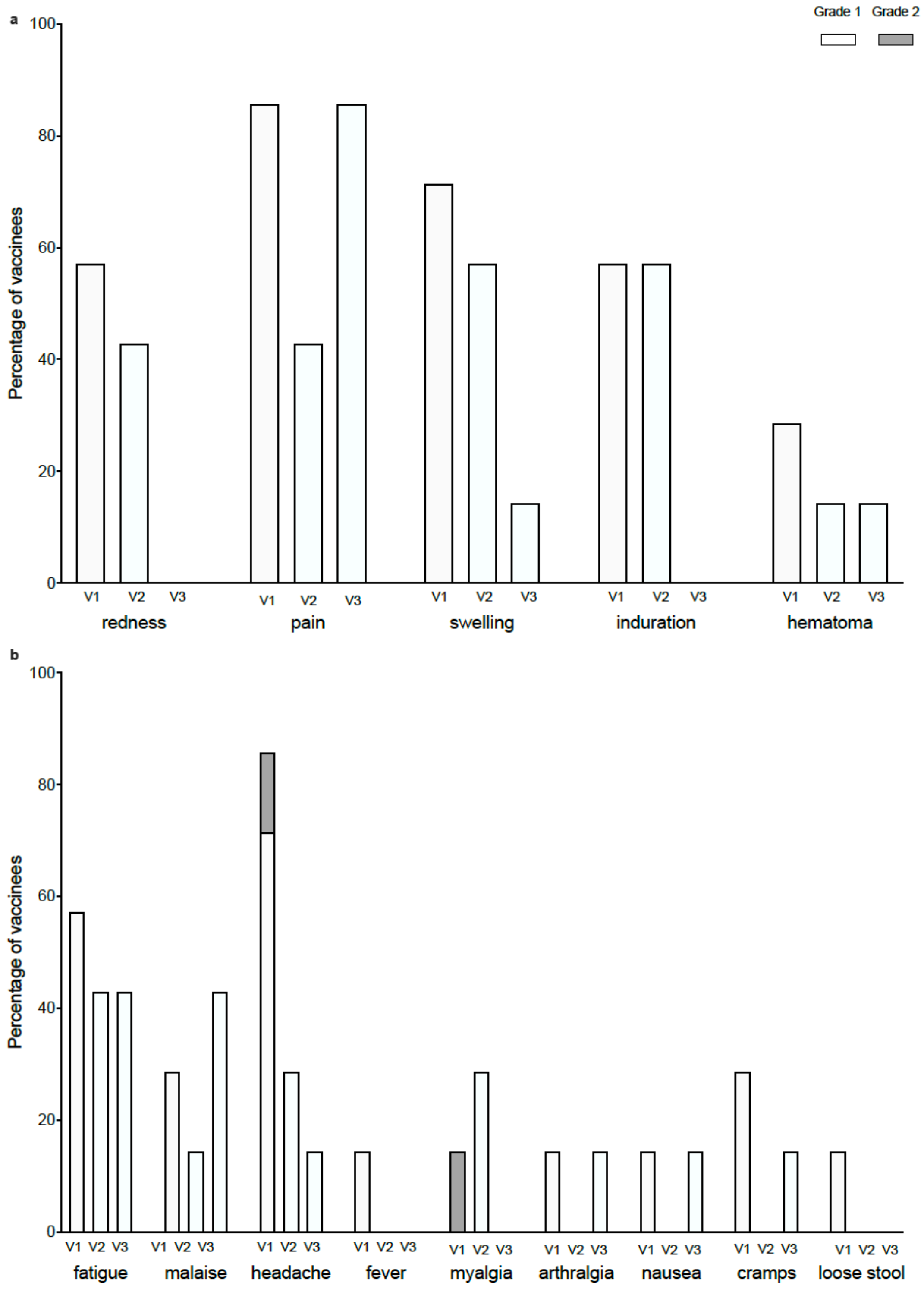

Percentage of participants in the former high dose cohort (n=7) who experienced solicited local (a) or systemic (b) adverse events (AEs) related to vaccination 1, 2 and 3 (V1, V2, V3). The frequency of Grade 1 (mild) and Grade 2 (moderate) AEs is depicted by white and gray bars, respectively. Grade 3 or higher AEs did not occur in this cohort. While the trial was not powered for statistical comparison, AE type, frequency and severity seemed to be comparable between vaccinations.

1.2 Supplementary Fig. 2. Relative changes of hematologic parameters and CRP after vaccination.

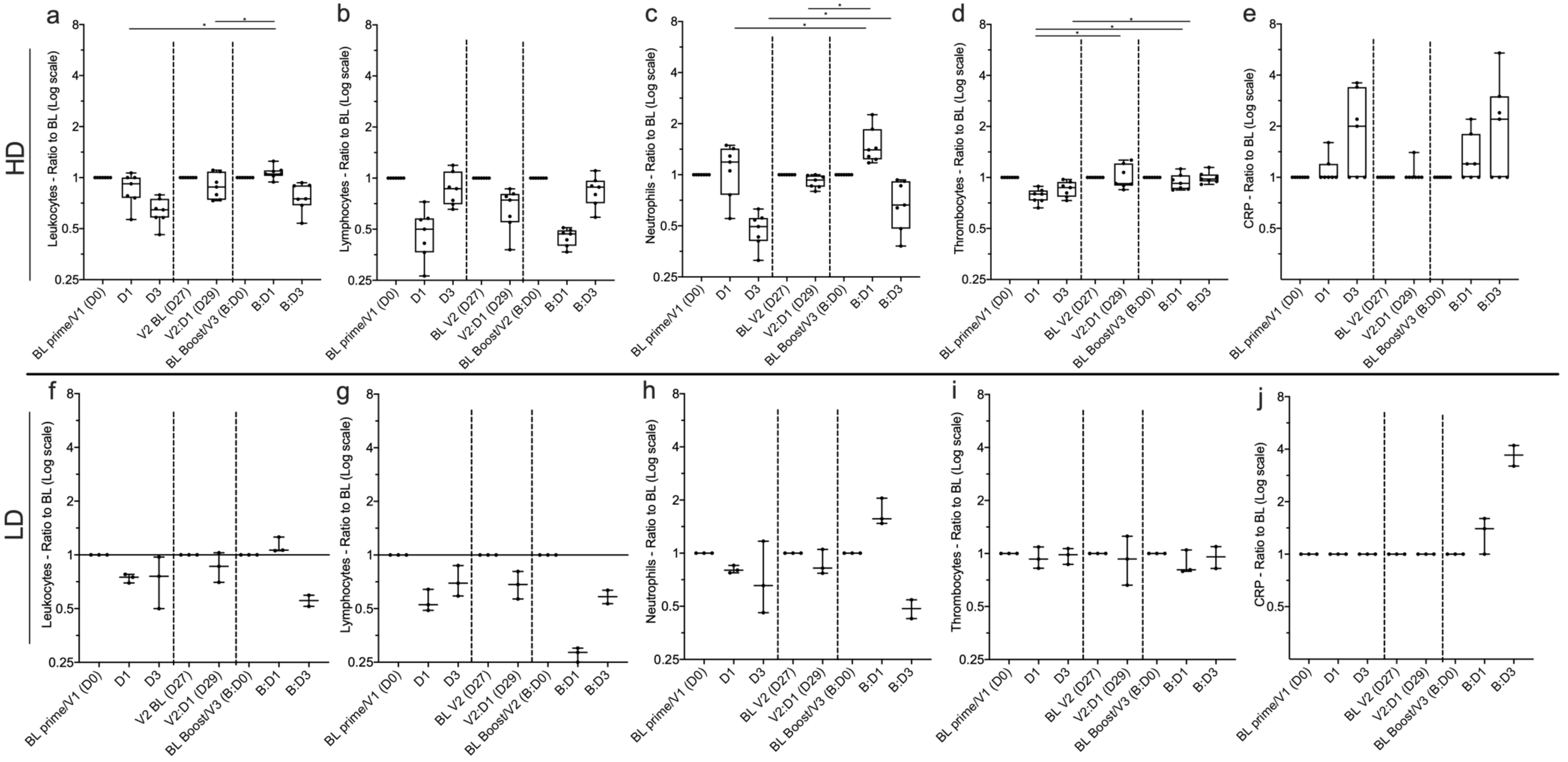

Relative changes from baseline parameters after prime (V1), second (V2) and booster vaccination (V3) in individuals who received $1x10^8$ plaque-forming units (PFU, n=7, high dose (HD), (a-e)) or $1x10^7$ PFU (n=3, low dose, LD (f-j)). (a-j) MVA-MERS-S as V1 and V2. The booster dose was $1x10^8$ PFU in all participants. Baseline (BL) was defined as the day before vaccination or pre-dose on vaccination day of each vaccination. Boxes indicate 25-75 percentile; whiskers are min. to max.; medians are shown as horizonal lines within the boxes. *p<0.05, specifically: (a) HD leukocyte changes: D1 vs. B:D1, p=0.047; V2:D1 vs. B:D1, p=0.016; (c) HD neutrophil changes: D1 vs. B:D1, p=0.031; D3 vs. B:D3, p=0.047, V2:D1 vs. B:D1, p= 0.016; (d) HD thrombocyte changes: D1 vs. B:D1, p=0.016; D1 vs. V2:D1, p=0.016; D3 vs. B:D3, p=0.047. Differences assessed using two-sided Wilcoxon matched-pairs signed rank test. Source data are provided as a Source Data file.

1.3 Supplementary Fig. 3. Correlation of neutralizing anti-MERS-CoV antibody levels measured by VNT.

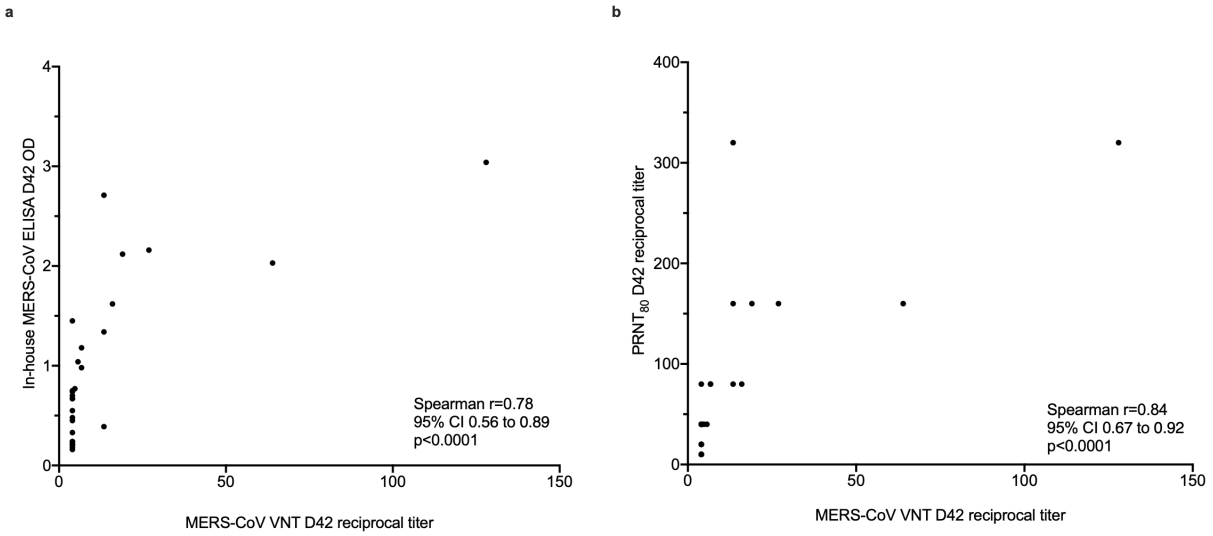

Correlation between anti-Middle East respiratory syndrome coronavirus (MERS-CoV) neutralizing antibody levels as measured by virus neutralization test (VNT) and (a) anti-MERS-CoV-S titers (in-house ELISA, measured in optical density (OD)) as well as (b) ≥80% plaque-reduction neutralization test ($PRNT_{80}$), both measured at Day 42 post prime vaccination show a strong inter-assay correlation ((a) r=0.78, 95% CI 0.56 to 0.89, p<0.0001, (b) r=0.84, 95% CI 0.67 to 0.92, p<0.0001), assessed by two-sided Spearman correlation, without adjustments for multiple comparison. N=29 pairs. Source data are provided as a Source Data file.

1.4 Supplementary Fig. 4. MERS-CoV-S peptide microarray including unvaccinated control.

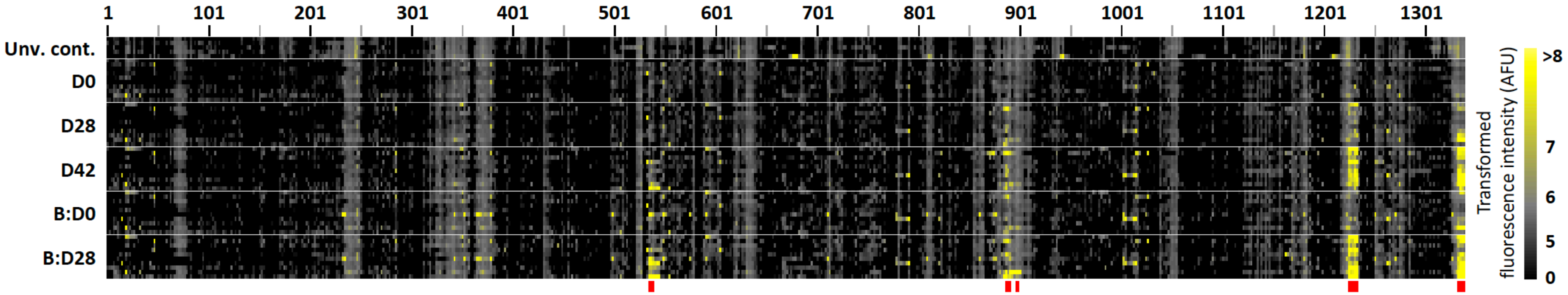

A microarray spanning the complete MERS-CoV-S protein with 15-mer peptides and a 13 AA overlap was utilized to assess IgG epitope binding of MERS-CoV-specific antibodies. Sera from all 10 vaccinees who received a booster vaccination were evaluated on Day (D) D0, D28, D42, Boost Day (B:D) B:D0 and B:D28 (one row per individual). In addition, serum from one unvaccinated control individual (Unv. cont.) drawn at the same timepoints was included as control (one row per timepoint). Epitope binding is depicted as transformed fluorescence intensity in arbitrary fluorescence units (AFU).

1.5 Supplementary Fig. 5. Longitudinal development of immunogenic anti-MERS-S IgG responses (RBD).

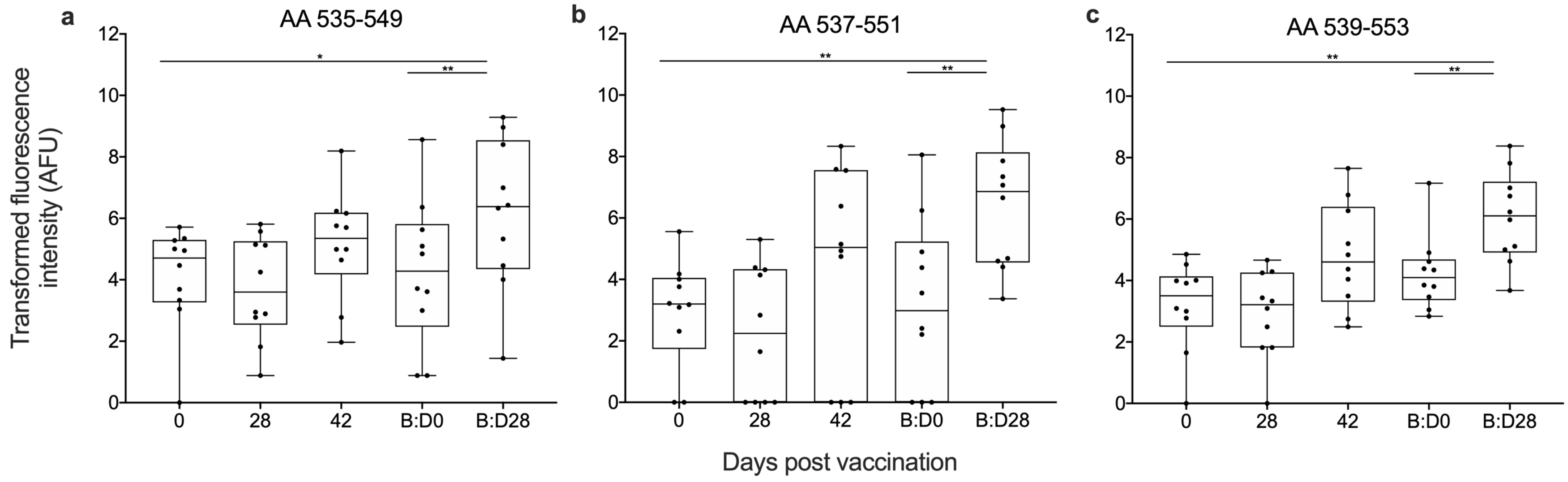

Longitudinal development of antibody responses to epitope amino acid (AA) 535-558, located in the receptor-binding domain (RBD) of MERS-CoV spike glykoprotein, measured as fluorescence intensity on IgG peptide microarrays and illustrated as transformed values of arbitrary fluorescence units (AFU) on Day 0, 28, 42, Boost Day (B:D) 0 and B:D28 in all individuals who received a booster vaccination (n=10). (a-c) depict the course of antibody generation against 15mer overlapping peptides constituting the epitope located at AA 535-553: (a) AA 535-549: `WEDGDYYRKQLSPLE` (D0 vs. B:D28, p=0.014; B:D0 vs.B:D28, p=0.004) ; (b) AA 537-551: `DGDYYRKQLSPLEGG` (D0 vs. B:D28, p=0.002; B:D0 vs.B:D28, p=0.004); (c) AA 539-553: `DYYRKQLSPLEGGGW` (D0 vs. B:D28, p=0.002; B:D0 vs.B:D28, p=0.004). Boxes indicate 25-75 percentile; whiskers are min. to max.; medians are shown as horizonal lines within the boxes. *p<0.05, **p<0.005, differences assessed using a two-sided Wilcoxon matched-pairs signed rank test. Source data are provided as a Source Data file.

1.6 Supplementary Fig. 6. Longitudinal development of immunogenic anti-MERS-S IgG responses (stem helix).

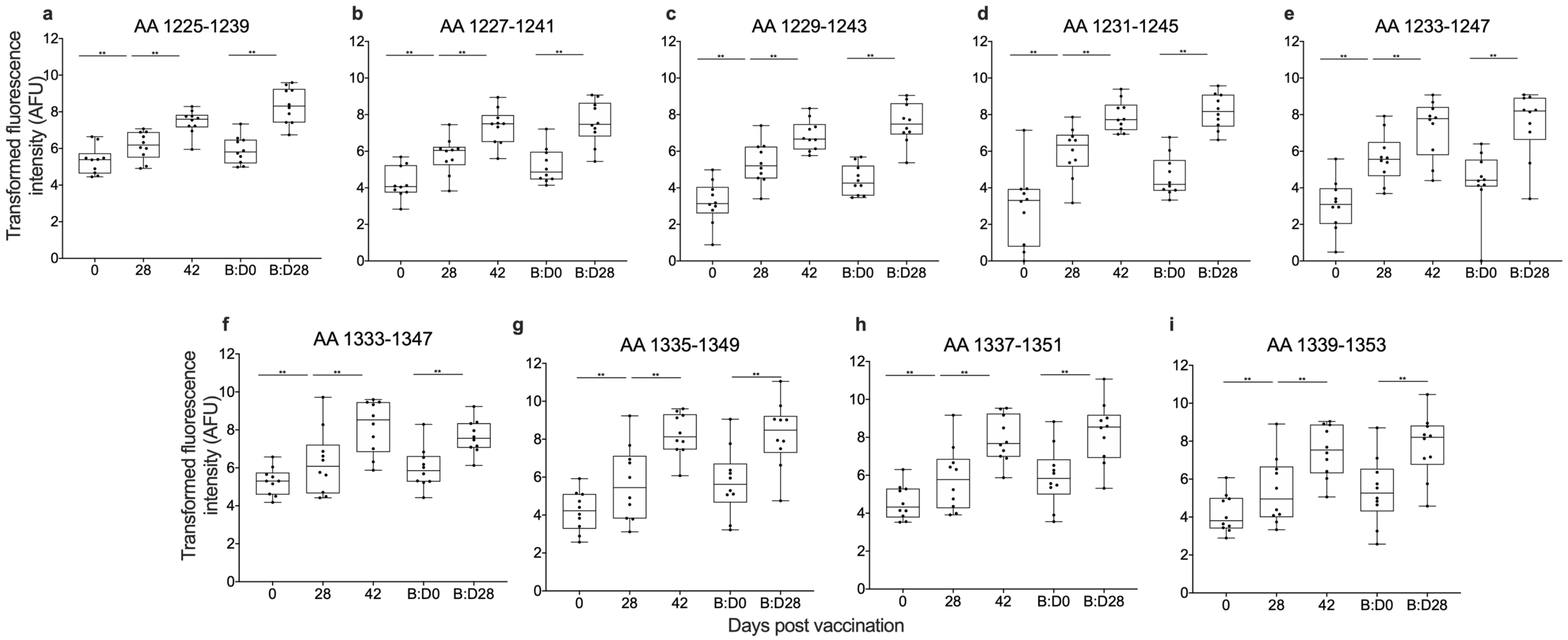

Longitudinal development of antibody responses to epitopes located in the stem helix of MERS-CoV-S measured as fluorescence intensity on IgG microarrays and illustrated as transformed values of arbitrary fluorescence units (AFU) on Day 0, 28, 42, Boost Day (B:D) 0 and B:D28 in all individuals who received a booster vaccination (n=10). Graphs (a-e) depict the course of antibody generation against 15mer overlapping peptides constituting the epitope located at amino acid (AA) 1225-1247: (a) AA 1225-1239: NSTGIDFQDELDEFF (D0 vs. D28, p=0.002; D28 vs. D42, p=0.002; B:D0 vs. B:D28, p=0.002); (b) AA 1227-1241: TGIDFQDELDEFFKN (D0 vs. D28, p=0.002; D28 vs. D42, p=0.002; B:D0 vs. B:D28, p=0.002); (c) AA 1229-1243: IDFQDELDEFFKNVS (D0 vs. D28, p=0.002; D28 vs. D42, p=0.002; B:D0 vs. B:D28, p=0.002); (d) AA 1231-1245: FQDELDEFFKNVSTS (D0 vs. D28, p=0.002; D28 vs. D42, p=0.002; B:D0 vs. B:D28, p=0.002); (e) AA 1233-1247: DELDEFFKNVSTSIP (D0 vs. D28, p=0.002; D28 vs. D42, p=0.001; B:D0 vs. B:D28, p=0.002), (f-i) represent the peptides constituting the AA 1333-1353 epitope: (f) AA 1333-1347: CNRCCDRYEEYDLEP (D0 vs. D28, p=0.001; D28 vs. D42, p=0.004; B:D0 vs. B:D28, p=0.002); (g) AA 1335-1349: RCCDRYEEYDLEPHK (D0 vs. D28, p=0.002; D28 vs. D42, p=0.002; B:D0 vs. B:D28, p=0.004); (h) AA 1337-1351: CDRYEEYDLEPHKVH (D0 vs. D28, p=0.002; D28 vs. D42, p=0.002; B:D0 vs. B:D28, p=0.002); (i) AA 1339-1353: RYEEYDLEPHKVHVH (D0 vs. D28, p=0.002; D28 vs. D42, p=0.004; B:D0 vs. B:D28, p=0.002). Boxes indicate 25-75 percentile; whiskers are min. to max.; medians are shown as horizonal lines within the boxes. *p<0.05, **p<0.005, differences assessed using a two-sided Wilcoxon matched-pairs signed rank test. Source data are provided as a Source Data file.

1.7 Supplementary Fig. 7. Correlation of anti-MERS-S antibody titers measured by IFA to neutralizing antibody levels.

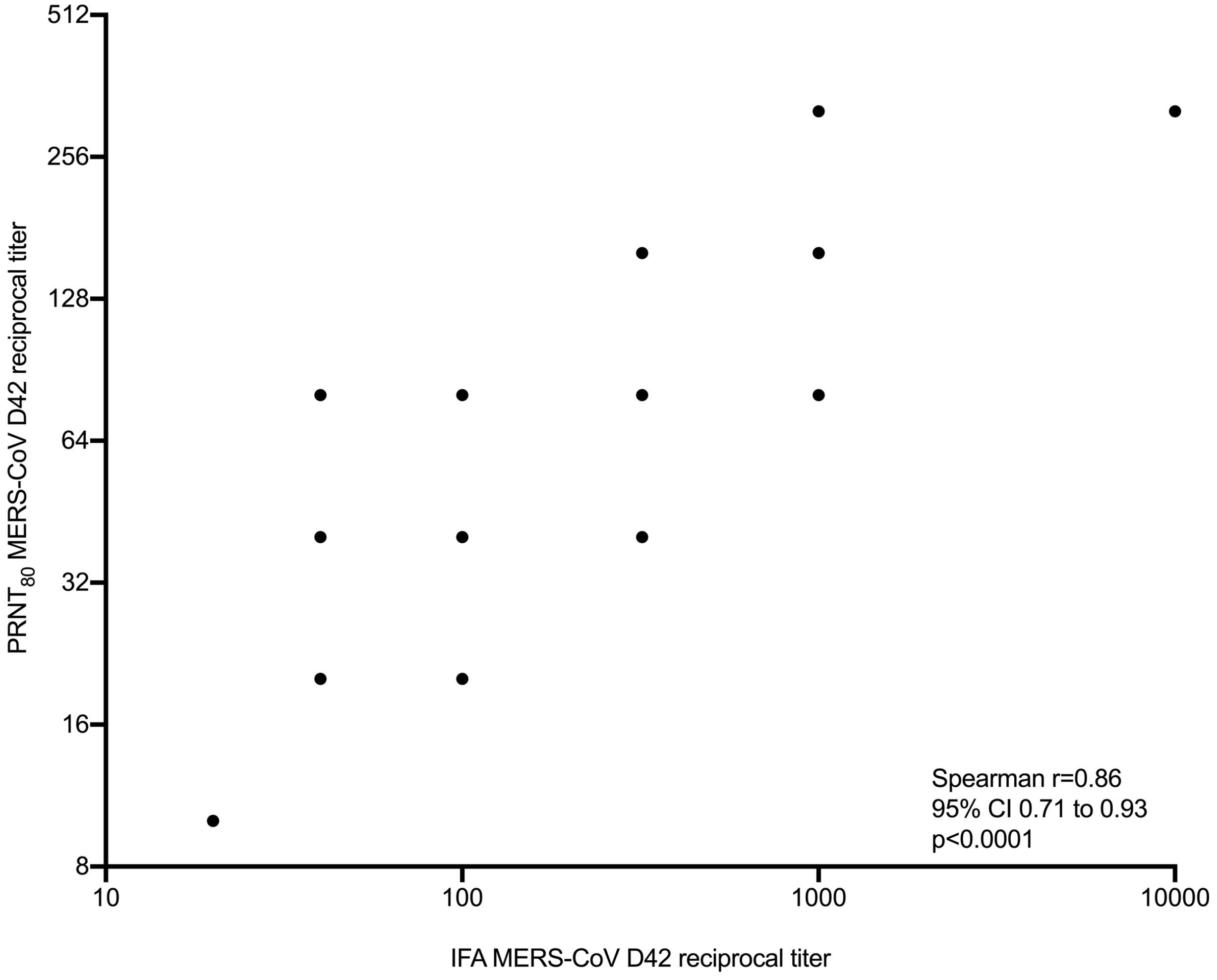

Neutralizing antibody levels as measured by ≥80% plaque reduction neutralization test ($PRNT_{80}$) and anti-Middle East respiratory syndrome coronavirus (MERS-CoV) spike glycoprotein-specific antibody titers measured by immunofluorescence assay (IFA) on Day 42 post prime vaccination show a strong inter-assay correlation (r=0.86, 95% CI 0.71 to 0.93, p<0.0001), assessed by two-sided Spearman correlation, without adjustments for multiple comparisons. N=29 pairs. Source data are provided as a Source Data file.

2 TABLES

2.1 Supplementary Table 1. Baseline characteristics of study participants.

	**All participants late booster subgroup (n=10)**	**Control cohort (n=4)**
**Sex**		
**Female**	10 (100%)	2 (50%)
**Male**	0	2 (50%)
**Age** (years)		
Mean (SD)	29.0 (6.7)	35.3 (5.9)
Range	18 – 40	27 - 40
**Ethnicity**		
**- White**	10 (100%)	3 (75%)
**- Black or African American**	0	0
**- Native Hawaiian or Pacific Islander**	0	0
**- Hispanic/Latino**	0	0
**- American Indian/Alaska Native**	0	0
**- Asian**	0	1 (25%)
**BMI (kg/m^2)**		
**Mean (SD)**	23.1 (3.6)	22.9 (1.3)
**Range**	19.0 – 29.7	22.0 – 24.8
**SD=standard deviation. BMI=body-mass index. Sex, age and ethnicity were provided by the participants, BMI was assessed by investigators after measurement of height and weight at screening.**		

2.2 Supplementary Table 2. Frequency and severity of adverse events after late booster immunization.

AE severity	All vaccinees (*n*=10)	AE severity ct'd	All vaccinees (*n*=10)
Injection-site AE		Fatigue	
Pain		Grade 1	4/10 (40%)
Grade 1	9/10 (90%)	Grade 2	0
Grade 2 or 3	0	Grade 3	1/10 (10%)
Total	9/10 (90%)	Total	5/10 (50%)
Swelling		Malaise	
Grade 1	2/10 (20%)	Grade 1	4/10 (40%)
Grade 2 or 3	0	Grade 2	1/10 (10%)
Total	2/10 (20%)	Grade 3	2/10 (20%)
Hematoma		Total	7/10 (70%)
Grade 1	1/10 (10%)	Headache	
Grade 2 or 3	0	Grade 1	1/10 (10%)
Total	1/10 (10%)	Grade 2 or 3	0
Systemic Adverse Events		Total	1/10 (10%)
Abdominal discomfort/cramps		Arthralgia	
Grade 1	1/10 (10%)	Grade 1	2/10 (20%)
Grade 2	0	Grade 2	1/10 (10%)
Grade 3	1/10 (10%)	Grade 3	0
Total	2/10 (20%)	Total	3/10 (30%)
Nausea		Myalgia	
Grade 1	1/10 (10%)	Grade 1	1/10 (10%)
Grade 2 or 3	0	Grade 2	0
Total	1/10 (10%)	Grade 3	1/10 (10%)
Fever		Total	2/10 (20%)
Grade 1	0	Unsolicited systemic AE (2xdizziness, 1xnausea (D17))	
Grade 2	2/10 (20%)	Grade 1	3/10 (30%)
Grade 3	0	Grade 2 or 3	0
Total	2/10 (20%)	Total	3/10 (30%)
Chills		Unrelated AE	
Grade 1	3/10 (30%)	Grade 1	5/10 (50%)
Grade 2 or 3	0	Grade 2 or 3	0
Total	3/10 (30%)	Total	5/10 (50%)

2.3 Supplementary Table 3. Percentage of participants with positive immunofluorescence titers at baseline (Day 0).

Cohort	MERS-CoV	HCoV-NL63	HCoV-OC43	HCoV-229E	HCoV-HKU1	SARS-CoV
Low Dose	0% (0/12)	100% (12/12)	100% (12/12)	92% (11/12)	100% (12/12)	0% (12/12)
High Dose	0% (0/12)	100% (11/11)	100% (11/11)	100% (11/11)	100% (11/11)	0% (11/11)
Controls	0% (0/6)	100% (6/6)	83% (5/6)	67% (4/6)	100% (6/6)	0% (0/6)

2.4 Supplementary Table 4. Relationship between anti-HCoV-S baseline titers (D0) and anti-MERS-CoV-S titers on Day 42 in IFA.

**IFA titers**	anti-HCoV-OC43 D0	anti-HCoV-229E D0	anti-HCoV-HKU1 D0	anti-HCoV-NL63 D0
Anti-MERS-CoV D42 (Spearman r, (95% CI))	0.07 (-0.32-0.04)	-0.13 (-0.48-0.26)	-0.20 (-0.53-0.19)	0.02 (-0.36-0.39)
P value	0.734	0.517	0.302	0.937
Two-tailed p value, p ≤0.05 is considered significant. Number of pairs: 29				

2.5 Supplementary Table 5. Relationship between anti-HCoV-S and anti-MERS-CoV-S titer fold-change on Day 42 in IFA.

**IFA titers**	anti-HCoV-OC43 D42	anti-HCoV-229E d42	anti-HCoV-HKU1 D42	anti-HCoV-NL63 D42
Anti-MERS-CoV D42 (Spearman r, 95% CI)	-0.05 (-0.46-0.38)	-0.01 (-0.43-0.41)	0.03 (-0.40-0.45)	0.00 (-0.42-0.42)
P value	0.835	0.961	0.889	>0.99
Spearman correlation of anti-HCoV-S titer fold-change compared to anti-MERS-CoV-S titer fold-change on day 42 in IFA. Two-tailed p value. p ≤0.05 is considered significant.				

3. Study Protocol

Universitätsklinikum Hamburg-Eppendorf

STUDY PROTOCOL

An open, single center Phase I trial to assess the safety, tolerability and immunogenicity of two ascending doses of the candidate vaccine MVA-MERS-S

EudraCT No.	2014-003195-23
Protocol No.	UKE-DZIF1-MVA-MERS-S
Version/Date	6.0 / 27.11.2018
Sponsor	University Medical Center Hamburg-Eppendorf<br>Martinistr. 52<br>20246 Hamburg, Germany
Principal Investigator	Prof. Marylyn M. Addo, MD, PhD, MSc, DTM&H<br>University Medical Center Hamburg-Eppendorf<br>I. Department of Medicine<br>Martinistr. 52<br>20246 Hamburg, Germany<br><br>Telephone: [redacted]<br>Fax:<br>Email:

CONFIDENTIALITY STATEMENT
The information provided in the following document is confidential and is only available for review to investigators, potential investigators, the ethics committee and the competent authorities. No disclosure should take place without the written authorization from the sponsor, except to the extent necessary to obtain informed consent from potential subjects or to obtain approval of this protocol by an ethics committee or regulatory authorities.

1 SIGNATURES

This protocol has been approved by University Medical Center Hamburg-Eppendorf.

Sponsor Signature

Sponsor Signature	Date
Prof. Dr. Dr. U. Koch-Gromus	
Sponsor Name	

Principal Investigator

I hereby confirm that I have acknowledged the protocol and agree to conduct the study in compliance with the protocol.

Principal Investigator Signature	Date
Prof. Dr. med. Marylyn Addo	
Principal Investigator Name	

Statistician Signature

Statistician Signature	Date
Angelika Böhm	
Statistician Name	

Summary of Changes

Summary of changes since last version of approved protocol (protocol version 3.0 to version 4.0)		
**Amendment Number**	**Date of Amendment**	**Section Affected by Change**
01	20DEC2017	2 Synopsis
Brief description of change:<br>The synopsis was updated according to the information given in the main body.		
01	20DEC2017	6 Investigator and study administrative structure
Brief description of change:<br>Contact information was updated.		
01	20DEC2017	7.4 Risk-benefit considerations
Brief description of change:<br>Additional of information on applied risk management measures.<br>Total amount of blood draw corrected.		
01	20DEC2017	8.5 Exploratory Objectives
Brief description of change:<br>Clarification that the results from exploratory objectives may not be part of the final study report.		
01	20DEC2017	9.3.1 Inclusion Criteria
Brief description of change:<br>No. 4: Addition of tolerated non-clinically significant, minor deviations of laboratory measurements.<br>No. 7: Harmonization of information on the duration of using contraception.		
01	20DEC2017	9.3.2 Exclusion Criteria
Brief description of change:<br>No. 11: Deletion of migraines.		
01	20DEC2017	9.5.2.8.6 Cardiac biomarker
Brief description of change:<br>Correction of tube to be used for blood sampling for analysis of troponin t.		
01	20DEC2017	9.5.3.1 Immunogenicity Assays
Brief description of change:<br>Addition of reference to the laboratory manual for detailed information on the blood volume drawn for immunogenicity assays.		
01	20DEC2017	9.6.5 Archival of documents
Brief description of change:<br>Addition of information on the archival of the TMF.		
01	20DEC2017	9.7.1 Definition of Adverse Events, Period of Observation, Recording of Adverse Events
Brief description of change:<br>Clarification of time window for classification of an AE as *solicited*.		
01	20DEC2017	9.7.2 Severity Categorization of AEs
Brief description of change:<br>Clarification of details on AE grading.		
01	20DEC2017	9.8.1.3 Statistical analyses
Brief description of change:<br>Addition of possibility to perform interim analyses.		

Summary of changes since last version of approved protocol (protocol version 4.0 to version 5.0)		
**Amendment Number**	**Date of Amendment**	**Section Affected by Change**
02	06FEB2018	9.3.1 Inclusion Criteria
Brief description of change:<br>No. 4: Concretion of the tolerated non-clinically significant, minor deviations of laboratory measurements.		

Summary of changes since last version of approved protocol (protocol version 5.0 to version 6.0)		
**Amendment Number**	**Date of Amendment**	**Section Affected by Change**
03	27NOV2018	Synopsis
Brief description of change:<br>- 3rd vaccination 12 months after 1st immunization		
03	27NOV2018	Study schedule
Brief description of change:<br>Entering study schedule 1b and 2b to reflect the 3rd vaccination and the subsequent follow-up visits		
03	27NOV2018	5.3 Subject Information
Brief description of change:<br>Informed consent for study extension		
03	27NOV2018	6. Investigators and study administrative structure
Brief description of change:<br>Update of statistician		
03	27NOV2018	7.4.1.1 Phlebotomy
Brief description of change:<br>Increase of blood amount		
03	27NOV2018	9.3.1 Inclusion Criteria
Brief description of change:<br>No. 11 & 12 applicable contraceptive requirements for 3rd vaccination		
03	27NOV2018	9.3.2 Exclusion Criteria
Brief description of change:<br>No. 25: Receipt of any vaccine before and after 3rd vaccination		
03	27NOV2018	9.4.1 Treatment Administered
Brief description of change:<br>3rd vaccination was added		
03	27NOV2018	9.4.10.3.4 Ambulatory Visits
Brief description of change:<br>Adaption wording		
03	27NOV2018	9.4.10.3.5 Ambulatory Vaccination Visit
Brief description of change:<br>Ambulatory vaccination visit for 3rd vaccination		
03	27NOV2018	9.4.10.3.6. End-of-study Visit
Brief description of change:<br>End-of-study visit after 3rd vaccination		
03	27NOV2018	9.5.2.5 Vital signs
Brief description of change:<br>Deviation for vital signs for 3rd vaccination added		
03	27NOV2018	9.5.2.8.2 Hematology
Brief description of change:<br>Analysis of reticulocytes added		

03	27NOV2018	9.7.1 Definition of Adverse Events, Period of Observation, Recording of Adverse Events
Brief description of change:<br>List of solicited adverse events complemented/ specified in accordance with events documented in the subject diary		

2 SYNOPSIS

**Name of Sponsor/Company:** University Medical Center Hamburg-Eppendorf	**Individual Study Table Referring to Part of the Dossier**	**(For National Authority Use only)**
**Name of Finished Product:** MVA-MERS-S	**Volume:**	
**Name of Active Ingredient:** MVA-MERS-S	**Page:**	

**Title of Study:**	An open, single center Phase I trial to assess the safety, tolerability and immunogenicity of two ascending doses of the candidate vaccine MVA-MERS-S
**Principal Investigator**	Prof. Dr. med. M. Addo
**Study center:**	CTC North GmbH & Co. KG<br>at the University Medical Center Hamburg-Eppendorf<br>Martinistr. 64<br>20251 Hamburg, Germany
**Protocol-No.**	UKE-DZIF1-MVA-MERS-S
**EudraCT-No.**	2014-003195-23
**Study Period**	Approx. 7 months per patient (screening to follow-up)<br>Total study duration (FPFV to LPLV) approx. 13 months<br><br>For subjects who gave consent for a 3rd vaccination (= boost vaccination one year after 1st vaccination):<br>Approx. 10-18 months per patient (screening to follow-up)<br>Total study duration (FPFV to LPLV) approx. 18 months total duration
**Phase of development:**	Phase I, first-in-human
**Objectives:**<br>**Primary Objective**	• To evaluate the safety and tolerability of two dosage levels of the experimental vaccine MVA-MERS-S.<br>• To evaluate the reactogenicity after administration of two dosage levels of MVA-MERS-S
**Secondary Objectives**	• To evaluate MERS-S-specific antibody responses induced by two dosage levels of MVA-MERS-S
**Exploratory Objectives**	• To evaluate MERS-S -specific cellular immune responses after administration of MVA-MERS-S<br>• To characterize MERS-S -induced B and T cell memory responses<br>• To evaluate innate cell subset phenotypes and function induced by MVA-MERS-S<br>• To evaluate early innate immunity gene expression signatures induced by MVA-MERS-S<br>• To comprehensively investigate vaccine-induced humoral immune responses and antibody functions
**Study Design**	Single-center, open-label, ascending dose phase I study

**Name of Sponsor/Company:** University Medical Center Hamburg-Eppendorf	**Individual Study Table Referring to Part of the Dossier**	**(For National Authority Use only)**
**Name of Finished Product:** MVA-MERS-S	**Volume:**	
**Name of Active Ingredient:** MVA-MERS-S	**Page:**	

Methodology:

This will be a Phase I, single-center trial in 24 healthy adults aged 18 to 55 years.

Subjects will be allocated in two cohorts of 12 subjects each receiving two single vaccine injections 28 days apart. Vaccination of subjects in each cohort will proceed in a staggered manner.

Cohort	No. of vaccine injections	Dose per injection	No. of subjects
1	2	10^7 pfu	12
2	2	10^8 pfu	12

For subjects who gave consent for a 3rd vaccination (= boost vaccination one year after 1st vaccination):

Eligible subjects will receive a 3rd vaccination as a booster 12 months (±4 months) after the 1st vaccination.

Cohort	No. of vaccine injections	Dose per injection	No. of subjects
1	3	10^7 pfu<br>boost at 12 months:<br>10^8 pfu	max. 12
2	3	10^8 pfu	max. 12

<u>Safety considerations for dosing and dose-escalation:</u>

The study is designed to establish safety, tolerability and immunogenicity of MVA-MERS-S, a MERS Vaccine candidate, investigated at two different dose levels in 24 healthy adults in Germany.

An interim safety report, summarizing the safety (AEs, vital signs, 12-lead ECGs, and laboratory safety data) will be prepared and reviewed by the Principal Investigator and Medical Monitor before the second immunization for each dose cohort.

Planned dose escalation will occur after the second immunization of all subjects of the 1st dose group following a review of the interim safety

**Name of Sponsor/Company:** University Medical Center Hamburg-Eppendorf	**Individual Study Table Referring to Part of the Dossier**	**(For National Authority Use only)**
**Name of Finished Product:** MVA-MERS-S	**Volume:**	
**Name of Active Ingredient:** MVA-MERS-S	**Page:**	
	report summarizing the safety (AEs, vital signs, 12-lead ECGs, and laboratory safety data) and any available immunological data by the Principal Investigator and Medical Monitor.	
**Number of subjects:**	24 (12 per cohort)	
**Diagnosis and main criteria for inclusion:**	**Key inclusion criteria:**<br>• Written informed consent form<br>• Healthy male and female subjects aged 18-55 years.<br>• No clinically significant health problems as determined during medical history and physical examination at screening visit.<br>• Body mass index 18.5 - 30.0 kg/m^2 and weight > 50 kg at screening.<br>• Non-pregnant, non-lactating female with negative pregnancy test.<br>• Males and females who agree to comply with the applicable contraceptive requirements of the protocol as defined above from day 0 to day 56 (males) and 7 days prior vaccination to day 56 (females), respectively.<br>**Additional criteria for the subjects receiving the 3rd vaccination:**<br>• Males and females who agree to comply with the applicable contraceptive requirements of the protocol as defined above from day 0 to day 28 of the 3rd vaccination (males) and 7 days prior to 3rd vaccination to day 28 after the 3rd vaccination (females), respectively.<br><br>**Key exclusion criteria:**<br>• Receipt of any vaccine in the 2 weeks prior to 1st trial vaccination (4 weeks for live vaccines) or planned receipt of any vaccine in the 3 weeks following the 2nd trial vaccination.<br>• Previous MVA immunization.<br>• Known allergy to the components of the MVA-MERS-S vaccine product.<br>• Known history of anaphylaxis to vaccination or any allergy likely to be exacerbated by any component of the trial vaccine.<br>• Evidence in the subject's medical history or in the medical examination that might influence either the safety of the subject or the absorption, distribution, metabolism or excretion of the investigational product.<br>• Clinically relevant findings in ECG, in particular prolonged QTc (B) of > 450 msec in males and > 460 msec in females.	

**Name of Sponsor/Company:** University Medical Center Hamburg-Eppendorf<br>**Name of Finished Product:** MVA-MERS-S<br>**Name of Active Ingredient:** MVA-MERS-S	**Individual Study Table Referring to Part of the Dossier**<br>**Volume:**<br>**Page:**	**(For National Authority Use only)**
	• Evidence in the subject's medical history or in the medical examination that might influence either the safety of the subject or the absorption, distribution, metabolism or excretion of the investigational product under investigation.<br>• Any confirmed or suspected immunosuppressive or immunodeficient condition, cytotoxic therapy in the previous 5 years, and/or diabetes.<br>• Any chronic or active neurologic disorder, including seizures, and epilepsy, excluding a single febrile seizure as a child.<br>**Additional criteria for the subjects receiving the 3rd vaccination:**<br>• Receipt of any vaccine in the 2 weeks prior to (4 weeks for live vaccines) or 3 weeks following to 3rd trial vaccination.	
**Test product, dose and mode of administration, batch number:**	**Test product:** MVA-MERS-S<br>**Dose:**<br>• In Cohort 1, 12 fasted subjects will receive 2 single doses of 1 x 10^7 pfu_{50} MVA-MERS-S in 0.5mL (total injected volume 1mL).<br>• In Cohort 2, 12 fasted subjects will receive 2 single doses of 1 x 10^8 pfu_{50} MVA-MERS-S in 0.5mL (total injected volume 1mL).<br>• In addition, irrespective of cohort, eligible subjects that consent to participate in and are eligible for a 3rd boost vaccination will receive a 3rd single dose of 1 x 10^8 pfu MVA-MERS-S in 0.5mL (the total injected volume will then be 1.5mL).<br>The relative concentration of the test product is 2x10^7 pfu_{50} /ml and 2x10^8 pfu_{50} /ml, respectively.<br>Dose administration on day 0 and day 28 for each subject. Dose escalation will occur following a review of the interim safety report.<br>For eligible subjects, the 3rd vaccination will be administered 12 months after 1st vaccination ± 4 months.<br>**Mode of administration:**<br>Intramuscular (i.m.)	
**Duration of Treatment:**	Subjects will receive two single vaccine injections, one on day 0 and another on day 28.<br>In addition, eligible subjects will receive a boost vaccination 12 months (± 4 months) after the 1st vaccination.	

<table>
<tr><td>Name of Sponsor/Company:
University Medical Center Hamburg-Eppendorf</td><td rowspan="3">Individual Study Table Referring to Part of the Dossier

Volume:

Page:</td><td rowspan="3">(For National Authority Use only)</td></tr>
<tr><td>Name of Finished Product:
MVA-MERS-S</td></tr>
<tr><td>Name of Active Ingredient:
MVA-MERS-S</td></tr>
<tr><td>Reference therapy, dose and mode of administration, batch number:</td><td colspan="2">None</td></tr>
<tr><td>Criteria for evaluation:</td><td colspan="2">Primary Endpoints:
The nature frequency and severity of adverse events associated with MVA-MERS-S vaccine will be collected and measured as followed:
• Occurrence of solicited local reactogenicity signs and symptoms for 14 days after vaccination.
• Occurrence of solicited systemic reactogenicity signs and symptoms for 14 days after vaccination
• Occurrence of unsolicited adverse events (AE) for 28 days after vaccination
• Change from baseline of safety laboratory measures
• Occurrence of serious adverse events (SAE) throughout the study period

Secondary Endpoints:
Immunogenicity
• Humoral immunity: Magnitude of MVA-MERS-S antibody responses</td></tr>
<tr><td>Safety:</td><td colspan="2">Safety data (AEs, SAEs and Vital Signs) will be collected at all study visits.

Laboratory safety test (biochemistry, hematology and dipstick urinalysis) will be performed at screening, pre-dose and at study follow-up visit.</td></tr>
<tr><td>Statistical methods:</td><td colspan="2">Metric data will be summarized using descriptive statistics (number, mean, standard deviation, minimum, median and maximum). Categorical data will be summarized by using frequency tables (frequency and percent). It is not planned to test any hypotheses in a confirmatory sense.</td></tr>
</table>

STUDY SCHEDULES

Table 1a

**Phase of study**	**SCR**	**Confinement**			**Ambulatory Visits**			**Confinement**			**Ambulatory Visits**				**End of study**
**Study day**	**-28- -2**	**-1**	**0**	**1**	**3**	**7**	**14 (±2)**	**27**	**28**	**29**	**35**	**42 (±2)**	**56 (±3)**	**84 (±3)**	**180 (±7)**
Informed Consent	√														
I/E Criteria	√	√						√							
Medical History/ Demographics	√														
Update Medical History		√						√							
Physical Examination	√	√[a]						√[a]				√[a]			√
Height and Weight	√														√[c]
Vital Signs	√	√	√[d]	√	√	√	√	√	√[d]	√	√	√	√	√	√
12-lead ECG	√					√	√		√		√	√			
Ambulatory Visit	√				√	√	√				√	√	√	√	√
Confinement		√	√	√[e]				√	√	√[e]					
Dosing			√[i]						√[i]						
Injection site & systemic events/ reactions assessment			√[d]	√	√	√	√		√[d]	√	√	√			√
Temperature	√		√[d]	√	√	√	√		√[d]	√	√	√	√	√	√
Alcohol Breath Test	√	√						√							
Discharge				√						√					
Training/ Dispensing subject diary			√						√						
Review/ Collection of subject diary				√	√	√	√	√		√	√	√	√	√	√
Adverse Events & Concomitant medication			√[d]	√	√	√	√	√	√[d]	√	√	√	√	√	√
**Laboratory Analysis**															
**Phase of study**	**SCR**	**Confinement**			**Ambulatory Visits**			**Confinement**			**Ambulatory Visits**				**End of Study**
**Study day**	**-28- -2**	**-1**	**0**	**1**	**3**	**7**	**14 (±2)**	**27**	**28**	**29**	**35**	**42 (±2)**	**56 (±3)**	**84 (±3)**	**180 (±7)**
Troponin T	√					√	√		√		√	√			√
Safety Lab blood (Clinical Chemistry, Hematology)	√	√		√	√	√	√	√		√	√	√			√
Safety Lab Urine	√	√				√	√	√			√	√			√
HIV/HBV/HCV Serology	√														
Urine Drug Screen	√	√						√							
Pregnancy Test	√[h]	√[g]						√[g]							√[h]
Anti-MVA-MERS Elisa			√			√	√		√		√	√	√	√	√
Neutralization Assay			√			√	√		√		√	√	√	√	√
T-cell response			√			√	√		√[f]		√	√	√	√	√
RNA blood sample storage			√	√	√	√			√[f]	√	√				

Innate immunity			√	√	√	√			√	√	√				
PBMC freezing and plasma aliquots			√	√	√	√	√		√ f	√	√	√	√	√	√

Table 2b

3rd boost vaccination 8-16 months after initial vaccination

Phase of study	Phone Call	Dosing	Ambulatory Visits				End of study
**Study day**	**≥ - 7**	**0**	**1**	**3**	**7**	**14 (±2)**	**28(±2)**
Informed Consent		√[f]					
I/E Criteria		√[f]					
Update Medical History		√[f]					
Physical Examination		√[af]					
Weight		√[fc]					
Vital Signs		√[d]	√	√	√	√	√
12-lead ECG		√[f]					
Ambulatory Visit		√	√	√	√	√	√
Dosing		√[i]					
Injection site & systemic events/ reactions assessment		√[d]	√	√	√	√	√
Temperature		√[d]	√	√	√	√	√
Alcohol Breath Test		√[f]					
Training/ Dispensing subject diary		√					
Review/ Collection of subject diary			√	√	√	√	√
Adverse Events & Concomitant medication		√[d]	√	√	√	√	√
Phone call	√						
**Laboratory Analysis**							
**Phase of study**	**Phone Call**	**Dosing**	**Ambulatory Visits**				**End of Study**
**Study day**		**0**	**1**	**3**	**7**	**14 (±2)**	**28(±2)**
Troponin T		√					√
Safety Lab blood (Clinical Chemistry, Hematology)		√	√	√	√	√	√
Safety Lab Urine		√			√	√	√
Urine Drug Screen		√					
Pregnancy Test		√[fh]					√[h]
Anti-MVA-MERS Elisa		√			√	√	√
Neutralization Assay		√			√	√	√
T-cell response		√			√	√	√
RNA blood sample storage		√	√	√	√		

Innate immunity		√	√	√	√		
PBMC freezing and plasma aliquots		√	√	√	√	√	√

[a] Symptom-targeted physical examination

[b] Please refer to Table for frequency of vital signs examinations

[c] Only weight

[d] Please refer to Table for frequency of Adverse Events and Concomitant Medication questioning.

[e] Morning check-out

[f] Before immunization

[g] Serum pregnancy test

[h] Urine pregnancy test

[i] Subjects will be monitored and evaluated for AEs for at least 30 minutes after vaccination in attendance of an investigator

Table 2a: Detailed Time and Events

Study Day	Time after Dose administration (hours)	Dosing	Vital Signs	Temperature	Adverse Events/ Con Med	Injection site/ systemic reaction/ Events assessment
0	0[1]	√	√	√	√	
0	2		√	√	√	√
0	4		√	√	√	√
0	6		√	√	√	√
0	8		√	√	√	√
0	12		√	√	√	√
1	24		√	√	√	√
28	0[1]	√	√	√	√	√
28	2		√	√	√	√
28	4		√	√	√	√
28	6		√	√	√	√
28	8		√	√	√	√
28	12		√	√	√	√
29	24		√	√	√	√

[1] Events must be completed before dose administration

Table 2b: Detailed Time and Events for 3rd vaccination

Study Day	Time after Dose administration (hours)	Dosing	Vital Signs	Temperature	Adverse Events/ Con Med	Injection site/ systemic reaction/ Events assessment
0	0[1]	√	√	√	√	
0	2		√	√	√	√
0	4		√	√	√	√
0	6		√	√	√	√

3 TABLE OF CONTENTS

STUDY PROTOCOL ... 1
1 SIGNATURES ... 2
2 SYNOPSIS ... 6
3 TABLE OF CONTENTS ... 14
4 LIST OF ABBREVIATIONS ... 16
5 ETHICS ... 18
5.1 Independent Ethics Committee (IEC) or Institutional Review Board (IRB) ... 18
5.2 Ethical Conduct of the Study ... 18
5.3 Subject Information and Consent ... 18
5.4 Confidentiality ... 18
5.5 Insurance ... 18
5.6 Publication Policy ... 19
5.7 Qualification of the Investigator ... 19
6 INVESTIGATORS AND STUDY ADMINISTRATIVE STRUCTURE ... 20
7 INTRODUCTION ... 22
7.1 Background and Rationale ... 22
7.2 MERS Vaccine Development and Preclinical Studies Using the MVA-MERS-S Vaccine Vector ... 22
7.3 Clinical Experience available for MVA-MERS-S ... 23
7.4 Risk-benefit considerations ... 23
7.4.1 Potential Risks for Study Participants ... 23
8 STUDY OBJECTIVES ... 25
8.1 Primary Objective ... 25
8.2 Primary Endpoints ... 25
8.3 Secondary Objective ... 25
8.4 Secondary Endpoints ... 25
8.5 Exploratory Objectives ... 25
9 INVESTIGATIONAL PLAN ... 25
9.1 Overall Study Design and Plan: Description ... 25
9.2 Discussion of Study Design, Including the Choice of Control Groups ... 25
9.3 Selection of Study Population ... 26
9.3.1 Inclusion Criteria ... 26
9.3.2 Exclusion Criteria ... 26
9.3.3 Removal of Subjects from Therapy or Assessment ... 28
9.4 Treatments ... 30
9.4.1 Treatments Administered ... 30
9.4.2 Identity of Investigational Medicinal Product ... 31
9.4.3 Preparation and Administration ... 32
9.4.4 Method of Assigning Subjects to Treatment Groups ... 33
9.4.5 Selection of Doses in the Study ... 33
9.4.6 Selection and Timing of Dose for Each Subject ... 33
9.4.7 Blinding ... 33
9.4.8 Prior and Concomitant Therapy ... 33
9.4.9 Rescue Medication ... 33

9.4.10 Treatment Compliance 33
9.5 Study Procedures and Examinations 38
9.5.1 Screening Procedures 38
9.5.2 Study Examinations 38
9.5.3 Pharmacodynamic Measurements 42
9.5.4 Pharmacogenetic Measurements 45
9.6 Data Quality Assurance 45
9.6.1 Quality Assurance System 45
9.6.2 Monitoring 45
9.6.3 Documentation and Data Collection 45
9.6.4 Data Management 45
9.6.5 Archival of documents 46
9.7 Adverse Events 46
9.7.1 Definition of Adverse Events, Period of Observation, Recording of Adverse Events 46
9.7.2 Severity Categorization of AEs 47
9.7.3 Relationship Categorization 47
9.7.4 Outcome Categorization 47
9.7.5 Clinical Laboratory Evaluations 48
9.7.6 Abuse, Misuse, Overdose, and Medication Error 48
9.7.7 Serious Adverse Event (SAE) Procedures 48
9.7.8 Definition of serious adverse reaction (SAR) 49
9.7.9 Definition of suspected unexpected serious adverse reaction (SUSAR) 49
9.7.10 Regulatory Agency, Independent Ethics Committee, and Investigative Site Reporting 49
9.7.11 Assessment of Severity 49
9.8 Statistical Methods Planned in the Protocol and Determination of Sample Size 52
9.8.1 Statistical and Analytical Plan 52
9.8.2 Determination of Sample Size 52
9.9 Changes in the conduct of the study or planned analysis 52
9.10 Local Safety Board 53
10 REPORTS 53
10.1 Final Report 53
10.2 Additional reports 54
11 REFERENCES 55

4 LIST OF ABBREVIATIONS

ADR	Adverse Drug Reaction
AE	Adverse Event
ALP	Alkaline phosphatase
ALT	Alanine transaminase
AMG	Gesetz über den Verkehr mit Arzneimitteln (Arzneimittelgesetz)
AST	Aspartate transaminase
cm	Centimeter
CoV	Coronavirus
CRF	Case Report Form
CRO	Contract Research Organization
CRP	C reactive protein
CV	Coefficient of Variation
DMP	Data Management Plan
DRM	Data Review Meeting
DZIF	German Center of Infectious Diseases Research (Deutsches Zentrum für Infektionsforschung)
ECG	Electrocardiogram
EDTA	Ethylene Diamine Tetra-acetic Acid
ELISA	Enzyme-linked Immunosorbent Assay
EliSpot	Enzyme-linked Immunosorbent Spot
EU	European Union
FDA	Food and Drug Administration
FIH	First in human
FIM	First in Man
FOCP	Female of Child-bearing Potential
GCP	Good Clinical Practice
GCP-V	Regulations on the implementation of Good Clinical Practice in the conduct of clinical trials on medicinal products for human use (GCP Verordnung)
GeoM	Geometric Mean
GGT	Gamma glutamyl transferase
GLP	Good Laboratory Practice
H	Hour
HBV	Hepatitis B Virus
HCV	Hepatitis C Virus
i.m.	Intramuscular
IB	Investigator’s Brochure
ICH	International Conference on Harmonization of Technical Requirements for Registration of Pharmaceuticals for Human Use
ICS	Intracellular Cytokine Staining
ID	Identification
IEC	Independent Ethics Committee
IMP	Investigational Medicinal Product
IRB	Institutional Review Board
ISF	Investigator Site File

kg	Kilogram
KSA	Kingdom of Saudi Arabia
LDH	Lactate dehydrogenase
MAH	Marketing Authorization Holder
Max	Maximum
MCH	Mean corpuscular hemoglobin
MCHC	Mean corpuscular hemoglobin concentration
MCV	Mean corpuscular volume
MDRD	Modification of Diet in Renal Disease
Mean	Arithmetic mean
Med	Median
MedDRA	Medical Dictionary for Regulatory Activities
MERS	Middle East Respiratory Syndrome
MET	Methamphetamine
mg	Milligram
Min	Minimum
mL	Milliliter
mmHg	Millimeters of mercury
msec	Millisecond
MTDMVA	Modified Vaccine Ankara
MVA	Modified Vaccinia Virus Ankara
N	Number
NHP	Non-Human Primates
PBMC	Peripheral Blood Mononuclear Cell
PEI	Paul Ehrlich Institute
PFU	Plaque-forming Unit
PI	Principal Investigator
QAU	Quality Assurance Unit
RBC	Red blood cells
SAE	Serious Adverse Event
SAP	Statistical Analysis Plan
SAR	Serious Adverse Reaction
SARS	Severe Acute Respiratory Syndrome
SD	Standard Deviation
SDV	Source Data Verification
SmPC	Summary of Product Characteristics
SOP	Standard Operating Procedure
SUSAR	Suspected unexpected Serious Adverse Reaction
TCA	Tricyclic Antidepressants
THC	Tetrahydrocannabinol
TMF	Trial Master File
WBC	White blood cell
WHO	World Health Organization

5 ETHICS

5.1 Independent Ethics Committee (IEC) or Institutional Review Board (IRB)

This study will be planned and performed in accordance with

- The Declaration of Helsinki in its version of Fortaleza, 2013;
- The EU Clinical Trial Directive 2001/20/EC;
- The "Note for Guidance on Good Clinical Practice" (CPMP/ICH/135/95 of January 17, 1997);
- The German Drug Law (Arzneimittelgesetz), in the current version;
- GCP-Verordnung from August 9, 2004 (GCP-Regulation)

and other applicable laws.

5.2 Ethical Conduct of the Study

The sponsor authorizes CTC North to make all necessary applications.

CTC North will submit, among other documents, the study protocol, the subject information and the informed consent form to the 'Ethik-Kommission der Ärztekammer Hamburg' and request approval (favorable opinion). CTC North will provide the clinical trial application to the competent authority (PEI). CTC North will notify the beginning (first subject's screening) and the end (last subject's last visit) of this trial and all amendments to the local Hamburg authority in accordance with § 67 Abs. 1 AMG.

The approval of both, the ethics committee and the PEI must be obtained prior to the start of the study. Copies of the original approval documents will immediately be sent to the sponsor and will be included in the study report. A list of the members of the IEC will also be provided with the final report.

5.3 Subject Information and Consent

An investigator will explain to the subjects the nature, significance and implications of the study prior to the clinical examination. The investigator will explain all methods, rules of conduct and any restrictions which may apply. Possible effects and side effects will be discussed. Subjects will be informed that they are free to withdraw from the study at any time, without giving any reason for doing so. They must be able to understand the full implications of their decision.

All participants will sign an informed consent form as evidence of consent. The subject information sheet and the informed consent form of each participant will be filed in the ISF. A second original of the signed consent form and a copy of the information sheet will be handed to the subjects after signature and before enrollment.

All participants will be informed about the extension of the study and the 3rd vaccination. Subjects willing to participate in a 3rd vaccination as a one-year boost after the initial vaccination, will be informed about the nature, significance and implications of the additional boost as part of this study by an investigator in a separate meeting prior to the clinical examination. The investigator will explain all methods, rules of conduct and any restrictions which may apply. Possible effects and side effects of the 3rd vaccination will be discussed and all subjects will sign the current version of the informed consent form as evidence of their consent.

5.4 Confidentiality

The investigator must assure that subjects' anonymity will be strictly maintained and that their identities are protected from unauthorized parties. Only an identification code (i.e., consists of identification number, sex and year of birth) should be recorded on any form or biological sample submitted to the laboratory, sponsor or IEC. The investigator must keep a screening log showing codes and names for all subjects screened and for all subjects enrolled in the trial.

5.5 Insurance

The sponsor is responsible for the appropriate insurance coverage for the subjects. Sponsor will take out insurance coverage for the subjects before regulatory submission, and the insurance certificate will be filed in the trial master file (TMF).

5.6 Publication Policy

The sponsor has to publish the result of this study in accordance with § 42b AMG. Beside it is in the sole discretion of the sponsor whether or not to publish the results of this study. The trial will be registered at clinicaltrials.gov.

5.7 Qualification of the Investigator

The Principal Investigator (PI) and his deputies fulfill the requirements of German law (§ 40 Abs. 1, Nr. 5 AMG). Curriculum vitae of the PI and deputies will be filed in the TMF.

For conducting the study the PI may delegate tasks to physicians (sub-investigators) or other qualified staff. This is to be documented properly. The PI is responsible for the adequate training and supervision of all delegates. No study related procedure must be performed by personnel which is not properly trained and delegated.

In the present document the mere term “Investigator” refers to the Principal Investigator or a sub-investigator.

6 INVESTIGATORS AND STUDY ADMINISTRATIVE STRUCTURE

**Sponsor:**	University Medical Center Hamburg-Eppendorf<br>Martinistr. 52, 20246 Hamburg, Germany
**Principal Investigator:**	Prof. Marylyn M. Addo<br>I. Med. Department<br>University Medical Center Hamburg-Eppendorf<br>Martinistr. 52, 20246 Hamburg, Germany<br>Phone: [redacted]<br>Fax: [redacted]<br>Email: [redacted]
**Deputy:**	Dr. med. Alen Jambrecina<br>CTC North GmbH & Co. KG<br>Martinistr. 64, 20251 Hamburg, Germany<br>Phone: [redacted]<br>Fax: [redacted]<br>Email: [redacted]
**Deputy:**	Dr. med. Stefan Schmiedel<br>I. Med. Department<br>University Medical Center Hamburg-Eppendorf<br>Martinistr. 52, 20246 Hamburg, Germany<br>Phone: [redacted]<br>Fax: [redacted]<br>Email: [redacted]
**Medical Monitor:**	Prof. Dr. med. Jan Rupp<br>Infectious Diseases<br>DZIF Clinical Trials Unit<br>University of Lübeck
**Clinical CRO**	CTC North GmbH & Co. KG<br>at the University Medical Center Hamburg-Eppendorf<br>Martinistr. 64, 20251 Hamburg, Germany
**Clinical Trial Management:**	Dr. Saskia Borregaard<br>CTC North GmbH & Co. KG<br>Phone: [redacted]<br>Fax: [redacted]<br>Email: [redacted]
**Project Manager:**	Laura Kaltenberg<br>CTC North GmbH & Co. KG<br>Phone: [redacted]<br>Fax: [redacted]<br>Email: [redacted]

Clinical Monitor: Ullrich Twisselmann
CTC North GmbH & Co. KG

Data Manager: Noelia Carabelos
CTC North GmbH & Co. KG
Phone: [redacted]
Fax: [redacted]
Email: [redacted]

Statistician: Angelika Böhm
Staburo GmbH
Aschauer Str. 30a, 81549 München, Germany
Phone: [redacted]
Fax: [redacted]
Email: [redacted]

Safety Laboratory Center for Diagnostics
University Medical Center Hamburg-Eppendorf
Martinistr. 52, 20246 Hamburg
Germany
Phone: [redacted]
Fax: [redacted]

Immunogenicity Laboratories:

**Humoral Immunity / Antibody responses**	**Cellular and Innate Immunity**
Prof. Stephan Becker<br>Institute for Virology<br>Philipps University Marburg<br>Hans Meerweinstr. 2<br>35043 Marburg, Germany<br>Phone: [redacted]<br>Fax: Email	Prof. Marylyn M. Addo<br>PD Julian Schulze-zur-Wiesch<br><br>ZIM, 1. Medizinische Klinik, UKE<br>c/o Heinrich-Pette-Institut<br>Leibnitz-Institut für Experimentelle Virologie<br>Martinistr. 52, 20251 Hamburg, Germany<br>Phone: [redacted]<br>Fax:<br>Mobile:<br>Email:
**Humoral Immunity / Antibody responses**	**TCR and Cellular Immunity**
Prof. Florian Klein<br>Labor für Experimentelle Immunologie<br>Klinik I für Innere Medizin<br>Zentrum für Molekulare Medizin<br>Universität zu Köln<br>Robert-Koch-Str. 21, Gebäude 66,<br>50931 Köln, Germany<br>Phone: [redacted]<br>Email	Dr. Cesar Munoz-Fontela<br><br><br>Bernhard-Nocht-Institut für Tropenmedizin<br>Bernhard-Nocht-Straße 74<br>20359 Hamburg, Germany<br>Phone: [redacted]<br>Email: [redacted]

7 INTRODUCTION

7.1 Background and Rationale

MERS and the need for a MERS Vaccine

In the aftermath of the dramatic and unprecedented Ebola outbreak in West-Africa 2013-2016, for which the world and scientific community were ill-prepared, the WHO has initiated a Research and Development "Blueprint Initiative for action to prevent epidemics" (http://www.who.int/csr/research-and-development/blueprint/en/). In this context WHO convened an ad-hoc expert group to synthesize lessons learned from past global health experiences and agreed on a list of emerging pathogens likely to cause severe outbreaks and for which no or limited medical countermeasures exist (http://www.who.int/medicines/ebola-treatment/WHO-list-of-top-emerging-diseases/en/). MERS-CoV, the causative agent of the Middle Eastern Respiratory syndrome (MERS) is one of the eight priority organisms for which such preparedness efforts will be initiated through international collaboration and the current vaccine trial falls under this umbrella funded by the German Center of Infectious Diseases Research (DZIF).

MERS is a potentially fatal disease under tight epidemiologic control by the WHO and currently without registered prevention or treatment option. The first case of MERS-CoV infection was identified in a patient with acute pneumonia and renal failure in the Kingdom of Saudi Arabia (KSA) in June 2012 [1]. As of December 6, 2016 1842 laboratory-confirmed cases of MERS-CoV, more than 652 deaths and 27 affected countries have been registered by WHO, mainly in the middle east, but also South Korea, the United Kingdom, France and Germany [2]. Three cases have been imported to Germany, all of which fatal and recently in September 2016 the second case has been imported to Austria. Transmission of the virus is from man-to-man and from human contact with non-human sources of virus, potentially dromedary camels. The current hypothesis is that primary cases result from transmission from camels-to-humans and secondary transmission takes place from human-to-human in the nosocomial setting. The virus affects men and women at all ages with severe courses of disease. Up to 40 % lethality has been reported for patients with comorbidities. Disease severities induced by MERS-CoV infections vary from silent infections over mild respiratory symptoms to severe pneumonia with acute respiratory distress syndrome, sepsis and multi-organ failure. Some cases develop renal failure concurrently with respiratory failure [3]. The causative agent of MERS is a Betacoronavirus within the subfamily Coronaviridae, closely related to SARS Coronavirus [4]. The spike surface glycoprotein S of MERS is responsible for binding to the human host cell receptor Dipeptidyl Peptidase IV, DPPIV [5], showing broad tissue distribution in humans (including bronchial and renal epithelia). Bat, civet, pig and rabbit DPPIV orthologs may support infection of corresponding cell lines, while canine, feline, rodent and chicken cells were found non-susceptible [6,7]. Importantly, a number of studies position the MERS-CoV spike protein S as a key target for virus neutralizing antibodies [8,9,10] including our own work [11].

Modified Vaccinia Virus Ankara (MVA) is a replication-deficient viral vector that holds great promise as a safe and efficacious vaccine platform. MVA can be engineered to encode one or more foreign antigens [12] and thus function as a multivalent vaccine, eliciting cell-based and humoral immune responses. The vector can be used at biosafety level 1 and has intrinsic adjuvant properties.

Modified Vaccinia virus Ankara recombinant virus expressing Middle East Respiratory Syndrome Coronavirus Spike protein (MVA-MERS-S) is an experimental vaccine which has been shown to induce neutralizing antibodies against Middle East Respiratory Syndrome Coronavirus (MERS-CoV) in a range of animal models. Facing the human pathogenicity of MERS-CoV with case-fatality rates in the 30-40% range and the unclear epidemiological situation of MERS in the Middle East and outside it is suggested to promote early clinical studies that evaluate the tolerability and immunogenicity of the vaccine candidate MVA-MERS-S in humans.

7.2 MERS Vaccine Development and Preclinical Studies Using the MVA-MERS-S Vaccine Vector

The experimental vaccine MVA-MERS-S has been studied in several animal models as outlined in the investigator's brochure (IB) (13). More specifical primary pharmacology data were obtained in the following models and studies:

- Mouse immunogenicity (Song F et al., 2013)
- Protection from MERS-CoV challenge in transiently tranduced hDPPIV mice (Volz A et al., 2015)

- Rat immunogenicity (as part of GLP compliant pivotal nonclinical toxicology study)
- Dromedary camels (Haagmans BL et al., 2016)

To date there is no appropriate non-human-primate model established for MERS, that models human disease in a satisfactory manner.

Table 4 of the investigator brochure summarizes the findings in these models that collectively describe MVA-MERS-S as a vaccine candidate with good immunogenicity, affording protection from virus challenge in two relevant animal models (mice and camels) without significant toxicity.

7.3 Clinical Experience available for MVA-MERS-S

To date, there has been no prior clinical trial experience with the MVA-MERS-S vaccine. This trial will be a first-in–human study for the investigational product MVA-MERS-S.

However, ample clinical experience with the licensed MVA vaccine (Imvanex/Imvamune, Bavaria Nordic, EMA licensing 2013) for protection against smallpox and multiple MVA-vaccine constructs for other infectious diseases (HIV, Influenza, Malaria, Tb, cancer, hepatitis C&B) is available and more than 6,500 patients and healthy volunteers were enrolled in the clinical trials listed in table 15 [13]. The MVA vector has been used extensively in diverse study populations including children, cancer patients and HIV and other immunocompromised individuals. No vaccine-related serious adverse event (SAE) has been reported therein and reported adverse events (AE) were all mild-to-moderate in nature and clearly dose-dependent.

All AEs reported were transient and resolved spontaneously.

7.4 Risk-benefit considerations

The participation in a phase I/ FIM study may not be of a therapeutic benefit to healthy subjects. Known risks related to the pharmacological properties of the investigational compound and/or study modalities are possible. To our best knowledge and judgement, the IMP seems to be safe and no severe side effects and no unacceptable adverse drug reactions (ADR) are expected with this study, as the used MVA vaccine vector has been extensively used in clinical trials for other infectious diseases threats encompassing in >6500 individuals, including children, cancer patients and immunocompromised hosts. The vaccine vector has had an excellent safety profile and unexpected adverse reactions conferred by the antigenic insert are not anticipated.

Preclinical data suggest a favorable safety and tolerability profile. All relevant preclinical studies have been conducted that are needed to start clinical development. Based on available information and the design of the study, the sponsor and the Investigator consider the trial to be ethically acceptable. The duration of confinement, the medical surveillance and the chosen time interval for the sequential dosing of each dosing cohort are considered adequate to ensure safety of the subjects. Special consideration was given to adequate spacing of dosing and frequent safety assessments as mandated and recommended by the regulatory authorities.

During protocol development, processes and data have been identified which are critical to ensure human subject protection and the reliability of trial results.

7.4.1 Potential Risks for Study Participants

7.4.1.1 Phlebotomy

The total amount of blood sampled from a subject will be max. 610 mL (575 mL for the safety and immunogenicity sampling, 35 mL for screening) over 7 months and additional 300 ml for the subjects receiving a 3rd immunization. No more than 500 mL will be drawn in 3 months. These drawn blood volumes should not compromise these otherwise healthy subjects, and are equivalent volumes to voluntary blood donations. There may be minor bruising, local tenderness, presyncopal symptoms associated with venipuncture, or phlebitis which may subsequently result in thrombosis. These events which will not be documented as AEs if they occur.

7.4.1.2 Administration of IMP (Study Vaccine)

Serious allergic reactions including anaphylaxis may occur and for this reason subjects will be inoculated in a clinical area where Advanced Life Support trained physicians, equipment and drugs are im-

mediately available for the management of any serious adverse reactions. Participants will be observed for 24 h after vaccination. Subjects will be monitored and evaluated for AEs for at least 30 minutes after vaccination in attendance of an investigator.

7.4.1.3 Potential Benefits for Subjects

The vaccine recipients may benefit from protection against future MERS outbreaks. However, this trial is the first administrations of MVA-MERS-S vaccine to humans. Therefore, to date the risks-benefit ratio remains unknown. Participants will be strongly advised to not consider themselves protected against MERS after vaccination at this stage of vaccine development.

7.4.1.4 Summary of Potential Risks for Study Participants

In summary, preclinical data suggest a favorable safety and tolerability profile. An extensive body of relevant preclinical studies have been conducted that are needed to proceed to Phase I clinical testing. Although potential risks are known, such as Myo-/pericarditis, Vaccinia rash, eczema vaccinatum, Post-vaccinial encephalitis, so far clinical trials on MVA have reported transient and spontaneously resolved adverse events and no serious ones. Based on available information and the design of the study, the sponsor and the PI consider the trial to be ethically acceptable. The duration of confinement, the medical surveillance and the chosen time intervals for the sequential dosing of the first, second and third subjects of each dosing cohort are considered adequate to ensure maximal safety of the subjects and have been established with guidance of the competent authority. Thus, taking into account the safety measures to minimize risks for study participants, the exposure of healthy subjects with MVA-MERS-S vaccine is justified since the potential risks and disadvantages for study participants are outweighed by the potential benefits for medical research, medical practice and eventually for individuals at risk of MERS.

8 STUDY OBJECTIVES

8.1 Primary Objective

This study is designed to assess the overall tolerability and safety of two ascending doses of the experimental MVA-MERS-S vaccine (10^7 pfu and 10^8 pfu) administered to healthy subjects and to evaluate the reactogenicity after administration of two dosage levels of MVA-MERS-S. Subjects will be allocated to two different dose cohorts each receiving two vaccine injections, one at day 0 and another on day 28.

8.2 Primary Endpoints

The safety, tolerability, and reactogenicity of the experimental vaccine MVA-MERS-S will be measured as followed:

- Occurrence of solicited local reactogenicity signs and symptoms for 14 days following the vaccination (see Table 3)
- Occurrence of solicited systemic reactogenicity signs and symptoms for 14 days following the vaccination (see Table 4 and Table 5)
- Occurrence of unsolicited AEs for 28 days following the vaccination
- Change from baseline of safety laboratory measures
- Occurrence of SAEs throughout the study period

8.3 Secondary Objective

- To evaluate the MVA-MERS-S-specific antibody responses induced by two dosage levels of MVA-MERS-S

8.4 Secondary Endpoints

- Humoral immunity: Magnitude of MERS-S-specific antibody responses as assayed by ELISA

8.5 Exploratory Objectives

- To evaluate MVA-MERS-S–specific cellular immune responses after administration of MVA-MERS-S
- To characterize MERS-S-induced B and T cell memory responses
- To evaluate innate cell subset phenotypes and function induced by MVA-MERS-S
- To evaluate early innate immunity gene expression signatures induced by the MVA-MERS-S
- To comprehensively investigate vaccine-induced humoral immune responses and antibody functions

The results from exploratory objectives may not be part of the final study report.

9 INVESTIGATIONAL PLAN

9.1 Overall Study Design and Plan: Description

This is an open, single-center phase I dose escalation trial in 24 healthy subjects of both sexes. Subjects will be allocated to two different dose cohorts each receiving two vaccine injections, one at day 0 and another on day 28. For safety reasons, subjects will be vaccinated in both cohorts in a staggered manner. Dosing of subjects will commence in dosing cohort 1 (1x10^7 pfu) followed by dosing cohort 2 (1x10^8 pfu). Dosing of cohort 2 will not start before both vaccinations and safety assessments of the first dose cohort are completed.

Second immunization and dose escalation will be performed following a review of the interim safety report by the Local Safety Board.

9.2 Discussion of Study Design, Including the Choice of Control Groups

In close coordination with the national regulatory authorities, the study design, including doses and schedule has been optimized to safely investigate this first-in human vaccine candidate. The study will

be a single center, open label, dose escalation study of the MVA-MERS-S candidate delivered by i.m. injection at two dose levels. In this small study with focus on safety and tolerability, blinding was deferred to ensure maximal safety and transparency.

9.3 Selection of Study Population

24 healthy male and female subjects, aged between 18 and 55 years will be enrolled in this clinical trial. The subjects will be recruited from the study center subject pool and public advertisement. Allocation to a certain treatment number (or subject number) will be done in successive order following screening and based on the subjects' availability.

9.3.1 Inclusion Criteria

The subject must not be enrolled before all inclusion criteria (including test results) are confirmed. Subjects meeting all of the criteria listed below will be included in the study:

1. Ability to understand the subject information and to personally sign and date the informed consent to participate in the study, before completing any study related procedures.
2. Provided written informed consent.
3. Healthy male and female subjects aged 18 – 55 years inclusive at the time of consent. The date of signing informed consent is defined as the beginning of the screening period. This inclusion criterion will only be assessed at the first screening visit.
4. No clinically significant health problems as determined during medical history and physical examination and clinical laboratory results at screening visit. Following laboratory parameters should be within normal limits: WBC, ANC, platelets. AST, ALT and ALP should be ≤ULN, Hb≥ LLN, Troponin T < 2-fold ULN, urine glucose should be negative, CrCL >60ml/min, urine protein ≤30mg/dl and total bilirubin should not exceed 1,5 x ULN. Non-clinically significant, minor deviations of laboratory measurements (except for ALT, AST which must be ≤ULN, CrCl which must be >60ml/min and Troponin T < 2-fold ULN) can be tolerated as long as they will not increase the risk of the individual having an adverse outcome from participating in this study as judged by the investigator. Deviations of laboratory measurements from the reference ranges of more than grade 1 according to Common Toxicity Criteria lead to an exclusion of the subject.
5. Body weight in defined relation to height. Body mass index 18.5 – 30.0 kg/m^2 and weight >50 kg at screening.
6. Non-pregnant, non-lactating female with a negative pregnancy test at screening and on day -1/day 27.
7. Females of child-bearing potential who agree to comply with the applicable contraceptive requirements of the protocol (9.3.2.1) from at least 7 days prior to vaccination until day 56 or females who are permanently sterilized (at least 6 weeks post-sterilization).
8. Males who agree to comply with the applicable contraceptive requirements of the protocol as defined above from day 0 through day 56.
9. Be willing to refrain from blood donation during the course of the study.
10. The subject is co-operative and available for the entire study.

Additional criteria for the subjects receiving the 3rd vaccination:

11. Males and females who agree to comply with the applicable contraceptive requirements of the protocol as defined above from day 0 to day 28 of the 3rd vaccination (males) and 7 days prior to 3rd vaccination to day 28 after the 3rd vaccination (females), respectively.
12. Non-pregnant, non-lactating female with a negative pregnancy test on day 0 of the 3rd immunization.

9.3.2 Exclusion Criteria

Subjects are excluded from the study if any of the following criteria are met at screening or at day -1:

1. Prior receipt of a MERS vaccine or MVA immunizations.
2. Receipt of any vaccine in the 2 weeks prior to 1st trial vaccination (4 weeks for live vaccines) or planned receipt of any vaccine in the 3 weeks following the 2nd trial vaccination.
3. Known allergy to the components of the MVA-MERS-S vaccine product as eggs, chicken proteins, and gentamycin or history of life-threatening reactions to vaccine containing the same substances.
4. Known history of anaphylaxis to vaccination or any allergy likely to be exacerbated by any component of the trial vaccine.
5. Participation in a clinical trial or use of an investigational product within 30 days or five times the half-life of the investigational drug -whichever is longer- prior to receiving the first dose within this study.
6. Evidence in the subject's medical history or in the medical examination that might influence either the safety of the subject or the absorption, distribution, metabolism or excretion of the investigational product under investigation.
7. Clinically relevant findings in ECG, in particular prolonged QTc (B) of > 450 msec in males and > 460 msec in females.
8. Any positive result for HIV1/2, HCV antibody or HBs antigen testing.
9. Any confirmed or suspected immunosuppressive or immunodeficient condition, cytotoxic therapy in the previous 5 years, and/or diabetes.
10. Subjects with inflammatory, infectious and neuroinflammatory underlying disease which could cause an expected impairment of the blood brain barrier such as meningitis, multiple sclerosis, epilepsy, or Alzheimer's disease.
11. Any chronic or active neurologic disorder, including seizures, and epilepsy, excluding a single febrile seizure as a child.
12. Known history of Guillain-Barré Syndrome.
13. Active malignancy or history of metastatic or hematologic malignancy.
14. Suspected or known alcohol and/or illicit drug abuse within the past 5 years.
15. Moderate or severe illness and/or fever >38°C within 1 week prior to vaccination.
16. Administration of immunoglobulins and/or any blood products within the 120 days preceding study entry or planned administration during the study period. Oral or parenteral immunosuppressant and immunomodulating agents (including interferons) should be discontinued at least 4 weeks prior to first injection.
17. History of blood donation within 60 days of enrollment or plans to donate within the treatment phase (until the 2nd vaccination).
18. Receipt of chronic (defined as more than 14 days) immune suppressants or other immune-modifying drugs within 6 months of study inclusion (screening).
    - For corticosteroids, this will mean prednisone, or equivalent, greater than or equal to 0.5 mg/kg/day.
    - Intranasal and inhaled steroids are allowed. Topical steroids are permitted provided they are not required to be applied to injection site.
19. Subjects with skin lesions close to the injection site or active oral lesions will be excluded.
20. Thrombocytopenia, contraindicating intramuscular vaccination based on investigator's judgment.
21. Subjects with a significant infection or known inflammation.
22. History of relevant cardiovascular disorders or evidence of hyper- (sitting blood pressure systolic >140 or diastolic >90 mmHg) or hypotension (sitting blood pressure systolic <90 or diastolic <40 mmHg) at screening.
23. Subjects who are known or suspected not to comply with the study directives.

24. Any other significant finding that in the opinion of the investigator would increase the risk of the individual having an adverse outcome from participating in this study.

Additional criteria for the subjects receiving the 3rd vaccination:

Receipt of any vaccine in the 2 weeks prior to (4 weeks for live vaccines) or 3 weeks following the 3rd vaccination.

9.3.2.1 Reproductive Potential

The study population includes female of child-bearing potential (FOCP). FOCP have to agree to comply with the applicable contraceptive requirements of the protocol as named below for the duration of the study or females who are permanently sterilized (at least 6 weeks post-sterilization).

Effective contraception is defined as a contraceptive method with failure rate of less than 1% per year when used consistently and correctly and when applicable, in accordance with the product label for example:

- Oral contraceptives, either combined or progesterone alone,
- injectable progesterone,
- implants of etenogestrel or levonorgestrel
- oestrogenic vaginal ring
- percutaneous contraceptive patches,
- intrauterine device or intrauterine system,
- male partner sterilization at least 6months prior to the female subject's entry into the study, and a monogamous relationship
- male condom combined with a vaginal spermicide (foam, gel, film, cream or suppository).
- male condom combined with a female diaphragm, either with or without a vaginal spermicide (foam, gel, film, cream, or suppository)

Condoms are to be used with the mentioned acceptable contraceptives.

9.3.2.2 Restrictions

Subjects will be asked to fast for at least 8 hours prior to dose administration in the morning of day 0 and 28 until 2 hours after vaccination.

9.3.3 Removal of Subjects from Therapy or Assessment

The study in its entirety may be discontinued prematurely by the PI or sponsor at any time (see below), and/or individual subjects may terminate their participation prematurely, or have their participation be terminated by the Investigator.

9.3.3.1 Withdrawal of Subject from the Study

The following circumstances may lead to discontinuation of the study by an individual subject who will then be recorded as a drop-out include but are not limited to the following:

- Withdrawal for personal reasons
- Adverse events necessitating withdrawal from the study
- Sudden incidence of diseases
- Circumstances in which the health of the subject would be endangered upon continued participation in the study
- Subject non-compliance with study requirements
- An AE, which requires discontinuation of the study involvement or results in inability to continue to comply with study procedures
- Significant protocol violation
- Lost to follow up

- Other (must be specified)

The reason for withdrawal will be recorded in the eCRF. If withdrawal is due to an AE, appropriate follow-up visits or medical care will be arranged, with the agreement of the subject, until the AE has resolved, stabilized or a non-trial related causality has been assigned. The Local Safety Board may recommend withdrawal of subjects.

At least 3 documented attempts must be made within 2 weeks to contact any subject lost to follow-up at any time point prior to the last scheduled office visit. One of the documented attempts must include a written communication (e.g. post mail, courier) with acknowledgement of receipt requested (e.g. certified mail, registered mail) sent to the subject's last known address, requesting that they return any unused investigational medicinal product and return to the study site for final safety evaluations.

A replacing subject will be assigned to the same administration sequence as the drop-out and to an ID number which is the drop-out number plus 100 (drop-out subject number 102 means replacer number 202). In case of drop out after first administration of study medication a final examination should be performed whenever possible.

9.3.3.2 Handling Subjects with Premature Study Discontinuation

In the absence of a medical contraindication or significant protocol violation, every effort will be made by the Investigator to keep the subject in the study. If a subject has to be withdrawn, all efforts will be made to complete and report the trial observations as thoroughly as possible and to follow safety of the subject as per protocol.

Subjects who withdraw or are withdrawn from the study prior to injection will be replaced. No follow-up of these subjects will be performed and no data will be analyzed. Subjects withdrawing or withdrawn within the first 7 days after second injection will be replaced, and their data will be analyzed in the safety population (see section 9.8.1.2). Subjects withdrawing or withdrawn after study day 35 will not be replaced, and their data will be analyzed in the safety population and the per protocol population (see section 9.8.1.2).

When a subject withdraws from the study before the planned end of the study period, all investigations scheduled for the end-of-study visit should be performed if the subject agrees. End-of-study evaluation will be completed at the time of the subject's withdrawal, with an explanation of the reason for this entered onto the respective "end-of-study" section of the eCRF as follows:

- Adverse event (specify)
- Death
- Protocol violation (specify)
- Medical condition (specify)
- Consent withdrawal, not due to AE
- Lost-to follow-up
- Other (specify)

9.3.3.3 Criteria for Termination of the Study (Holding Rules)

Safety holding rules will apply throughout the entire study period. Further injections will be discussed with the Local Safety Board. Should a holding rule be activated, the PI will inform the sponsor. The sponsor has to inform the competent authorities and the ethics committee within 15 days.

If the event(s) occur in the higher-dose cohort, the follow-up phase of the lower dose cohort will continue. The discontinuation of a holding rule should be communicated to all entities in the same manner and timeframe as described above.

The Local Safety Board review will consider:

- The relationship of the AE or SAE to the vaccine
- The relationship of the AE or SAE to the vaccine dose, or other possible causes of the event
- If appropriate, additional screening or laboratory testing for other subjects to identify those who may develop similar symptoms, and alterations to the current informed consent form will be discussed

All injected subjects will be followed for safety until resolution or stabilization (if determined to be chronic sequelae) of their AE. AE grading will be done according to FDA's 2007 voluntary guidance: (14) or according to CTCAE v4 (15). Please refer to Table 3- Table 5 for further details.

The holding rules are as follow:

- Solicited (expected) local adverse events:
    - If more than 25% of injections (minimum 2 individuals) are followed by Grade 3 solicited swelling or pain or Grade 4 redness beginning within 3 days after injection (day of injection and one subsequent day) and persisting at Grade 3 (swelling or pain)/4 (redness) for >48 – 72 hours
- Solicited (expected) systemic adverse events:
    - If more than 25% of injections (minimum 2 individuals) are followed by Grade 3 solicited systemic AE (or Grade ≥ 3 physical observations as defined above and in Section 9.7.11) beginning within 3 days after study injection (day of injection and one subsequent day) and persisting at Grade ≥ 3 for >48 – 72 hours
- Unsolicited (unexpected) adverse events:
    - If more than 25% of subjects (minimum of 2 individuals) develop a Grade ≥ 3 unsolicited AE (including laboratory AE and physical observations) that is considered probably or definitely related to injection and persists at Grade 3 for >48 – 72 hours
- A serious adverse event occurs that may be related to the IMP

9.3.3.4 Study Termination

The PI or sponsor may terminate the trial at any time. In the case of study termination, investigators will be informed of the procedures to be followed to ensure adequate consideration is given to the protection of the subject's safety.

The Investigator will be responsible for informing the sponsor of the trial's termination within 24 hours. The sponsor has to inform the competent authorities and the ethics committee within 15 days.

9.4 Treatments

9.4.1 Treatments Administered

All eligible subjects will be allocated to one of the two dose cohorts according to the assignment list. Each subject will receive two or three vaccine injections administered as i.m. injections in the deltoid region of the upper arm muscle.

For safety reasons dose administration in each dose level will be performed in four groups. Dose administration for the first subject at all dose levels will be vaccinated 48 hours prior to the second groups (second and third subject) of this dose level. The second and the third subject will be vaccinated in an interval of at least 5 min and after a period of at least 48 hours after the first subject to assess for immediate hypersensitivity reaction (within 60 min after the vaccination) or cytokine release (within 24 hours following the vaccination). The next group of four subjects can be vaccinated in parallel (in intervals of at least 5 min) at least 24 hours after the second group and at least 24 hours before the last group. The last group of the dose cohort including five subjects will be dosed on the same day in intervals of at least 5 min. Dosing events are supervised by an Investigator.

Figure 1: Dose administration groups

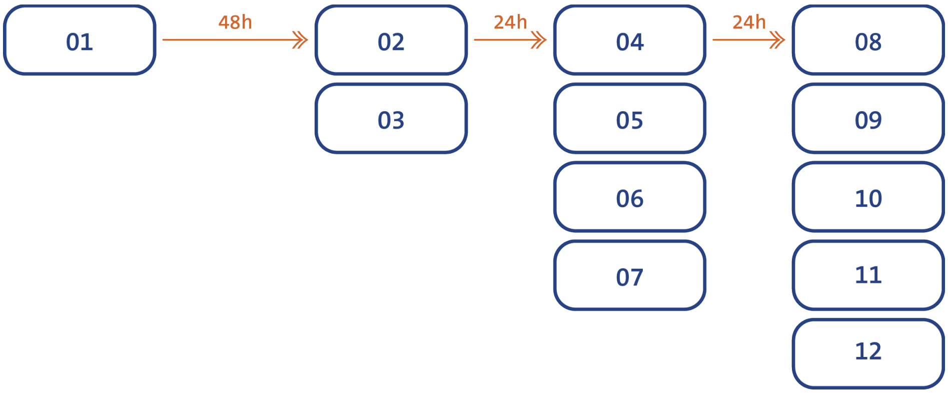

A Local Safety Board will be established for this study. Planned 2nd immunization and dose escalation will occur following a review of the interim safety report. Interim safety reports will contain safety data obtained until day 7.

The second immunization for each subject will be done after the Local Safety Board reviewed individual safety data of the respective subject.

All subjects who consented to the study extension will be immunized a third time with a vaccine dose of $1x10^8$ pfu MVA-MERS-S at 12 months (± 4 months) after the first vaccination.

For the decision about dose escalation safety data obtained until day 7 after the second immunization of all subjects of the first cohort will be taken into account.

A dose level may be repeated and furthermore, additional dose step(s) may be added but not exceeding dose of $1x10^8$ pfu MVA-MERS. The decision to repeat and/or to modify the dose level and/or to add additional dose steps will be taken by the Local Safety Board and the Ethics Committee and Competent Authority will be notified.

Repetition or modification of dose steps are only assumed for the second cohort if dose escalation is not possible due to safety-events observed within the lower dose cohort. Safety-events may be observed within the lower dose cohort that do not lead to study termination according to the holding rules but also do not warrant the increase of the dose up to $1x10^8$ pfu MVA-MERS. In this case, the second cohort may be vaccinated with the same dose as the first cohort or a dose lower than $1x10^7$ pfu MVA-MERS may be applied.

9.4.2 Identity of Investigational Medicinal Product

Name:	MVA-MERS-S
Dosage form:	Suspension for injection in a pre-filled syringe
Active substances:	MVA-MERS-S
Route:	IM injections
MAH:	Ludwig-Maximilians-Universität München

Additional information can be found in the Investigator’s Brochure [13].

9.4.2.1 Labeling

The study vaccine will be provided by IDT Biologika GmbH, Dessau to CTC North, and will be labeled in accordance with§ 5 GCP-V.

9.4.2.2 Packaging

Sponsor will provide study vaccine for this study. Investigational medicinal product will be provided in glass vials. These containers are suitable for the purpose and ensure the stability of the investigational medicinal product.

9.4.2.3 Storage

The investigator has overall responsibility for ensuring that study medication is stored under appropriate conditions in a secure, limited-access location. Study medication is distributed by a nominated member of the study team.

Study medication must be stored in accordance with labeled storage conditions (≤ -20°C). Temperature monitoring is required at the storage location to ensure that the study medication is maintained within an established temperature range. The temperature is monitored such that at least minimum and maximum temperature values throughout the total duration of the study are recorded and records are maintained.

The sponsor must be notified of any excursion from the established range. Relevant temperature excursions will require site investigation as to cause and remediation. The sponsor will determine the ultimate impact of excursions on the investigational medicinal product and will provide supportive documentation as necessary.

9.4.2.4 Shipment

The investigational medicinal products will be provided by the sponsor in sufficient quantity. The address for delivery purposes is:

CTC North GmbH & Co. KG
am Universitätsklinikum Hamburg-Eppendorf
Martinistr. 64
20251 Hamburg, Germany

The batch number of the IMP used in the study will be given in the study report.

9.4.2.5 Drug Accountability

The PI has the overall responsibility for administering the investigational medicinal products. The IMP must be administered in the manner specified in the study protocol and the pharmacy manual.

The PI or an authorized member of the study team will acknowledge receipt of the study medication documenting shipment content and condition. Damaged supplies will be replaced. Accurate records of all study medication received, dispensed, used, returned or destroyed must be maintained. No study medication may be destroyed or returned from the investigational site without prior knowledge and written consent by the sponsor. If such transfer is authorized by the sponsor, all applicable local, state and national laws must be adhered to for the transfer.

An Investigator will administer the investigational medicinal products. All administrations will be documented in the site's drug accountability log or other study drug record.

The sponsor or its representatives must be permitted access to review the supplies storage and distribution procedures and records.

At the end of the study or as instructed by the sponsor all unused stock and empty used boxes are sent to a nominated contractor or will be destroyed on behalf of the sponsor. Study medication being returned or destroyed must be counted and verified by clinical investigational site personnel and the sponsor or designee. All certificates of delivery/drug receipts should be signed by the site representative to confirm contents of shipment. Shipment return forms must be signed prior to shipment by CTC North. The sponsor must give authorization to return or destroy any study medication prior to shipment or destruction. Shipment of all returned study medication must comply with local, state, and national laws.

Based on the entries in the site drug accountability logs, it must be possible to reconcile study medication delivered with those used and returned. One hundred percent of the study medication must be accounted for and all discrepancies investigated and documented.

9.4.2.6 Subject Compliance

For study medication administration at CTC North, compliance must be assessed by observation of dosing. Details will be recorded on the drug accountability log or other appropriate source documents.

9.4.3 Preparation and Administration

Study medication will be provided in glass vials for administration with conventional syringes. Instructions for storage, preparation, administration, and destruction of study medication are presented in the pharmacy manual.

The vaccine will be injected intramuscularly into the deltoid region of the upper arm muscle.

The vaccine must not be administered to subjects allergic to the active substance, to any of the excipients, to eggs, chicken proteins, and gentamycin or with a history of life-threatening reactions to the study vaccine containing the same substances. Vaccine must not be administered to subjects with febrile illness or acute infection.

The study personnel must clean the site on injection with a suitable antiseptical and let the antiseptical evaporate before vaccination.

Subjects must be kept under observation by authorized members of the study team for 24 hours after vaccination to ensure their safety. Any reactions reported during observation period will be documented.

9.4.4 Method of Assigning Subjects to Treatment Groups

Subjects willing and eligible to participate in this study will be assigned to a treatment cohort according to the assignment list. Assignment to the 2nd dose cohort will start after all subjects for the 1st dose cohort are enrolled. The detailed process of subject identification and assignment of subject numbers can be found in section 9.4.10.2. No randomization is planned for this study.

9.4.5 Selection of Doses in the Study

The doses selected in this study represent immunogenic dose levels as observed in prior clinical trials using MVA-vector-based vaccines.

9.4.6 Selection and Timing of Dose for Each Subject

All dosing events will occur between 8:00 h and 14:00 h.

9.4.7 Blinding

As this is an open-label study, no blinding procedures are necessary.

9.4.8 Prior and Concomitant Therapy

As only healthy subjects will be enrolled, concomitant use of any medicinal product is forbidden prior to dosing as defined in section 9.3.2 *Exclusion Criteria* of this protocol. Prior to the dosing, subjects will be asked about this point.

Any use of drugs will be documented specifying the substance, dose, time and reason for use.

9.4.9 Rescue Medication

For symptomatic management of flu-like symptoms, the use of anti-pyretic drugs (e.g. paracetamol/ibuprofen) within the daily recommended dosage will be allowed after consultation of the investigator.

9.4.10 Treatment Compliance

9.4.10.1 Admission to the study

Admission to the study will be effective upon the subject's arrival at the study ward. A subject will only be admitted to the study if all inclusion and none of the exclusion criteria are met. Should there be any doubts as to the state of health, a subject will not be admitted to the study.

Subjects who fail to make themselves available upon commencement of the study or who cannot participate for personal reasons will be considered as not admitted to the study. These will be replaced by back-up subjects who will be recruited in surplus.

9.4.10.2 Subject Identification

Screening numbers are to be assigned sequentially to all subjects as they consent to take part in the study. This will be a 3-digit number starting at 001. The subject number is assigned to subjects when they are enrolled into the study and according to appearance of the subjects. This will be a 3-digit number starting at 101. For screening failures, the screening number will be the identifying number used throughout the documentation. Subjects, who will replace a subject e.g. a withdrawn subject, will receive a screening number according to the above described scheme. In difference to the described subject number assignment, replacement subjects will receive a 3-digit subject number which will consist of the old subject number (of the subject which should be replaced) plus 100.

Allocation to a certain subject number will be done in successive order following screening and based on the subjects' availability.

The investigator will keep a record relating the subject numbers and the names of all subject (including the CTC North identification number) that have given their informed consent, to allow easy checking of data in subject files, when required. This record will also include the date of subject's enrolment and completion, as well as subjects who could not be included in the study for whatever reason.

9.4.10.3 Screening, Confinement and Ambulatory Visits

9.4.10.3.1 Confinement (Day -1 to Day 1 and Day 27 to Day 29)

The Subjects will be admitted to the study ward in the evening on Day -1 going through regular check-in procedures including an ID check, vital signs, an inclusion/exclusion check, an alcohol breath test and a urine drug, and pregnancy test. The Subjects will stay on the ward until the morning of Day 1 experiencing blood draws for immunogenicity analysis, documentation of occurrence of any injection site reaction, systemic events/reaction and adverse event questioning throughout the confinement period. Subjects will be dismissed at the earliest after the 24 h after vaccination and after subjects were trained on the use of the diary.

This schedule will be repeated in for the second vaccination on day 27 to day 29.

9.4.10.3.2 Screening

At the screening visit the following interventions will be performed:

- Informed Consent
- I/E Criteria
- Medical History/ Demographics
- Physical Examination
- Vital Signs/Temperature
- ECG
- Height and Weight
- Alcohol Breath Test
- Urine Drug Screen
- Urine Pregnancy Test
- HIV/HBV/HVC Serology
- Safety Laboratory (Urine/Blood)
- Troponin T

9.4.10.3.3 Confinement Day -1 to Day 1 and Day 27 to Day 29

During confinement the following interventions will be performed:

- I/E Criteria check
- Update medical history
- Targeted physical examination
- Vital signs / Temperature
- 12-lead ECG (only on day 28)
- Injection Site reaction & systemic events/reaction assessment
- AE/ConMed
- Alcohol Breath Test
- Urine Drug Screen

- Serum Pregnancy Test
- Safety Laboratory (Urine/Blood)
- Blood sampling for humoral immunogenicity
- Blood sampling for cellular response
- PBMC freezing and plasma aliquots
- Blood sampling for RNA storage (only on day 0, 1, 28, 29)
- Blood sampling for troponin (only on day 28)
- Blood sampling for innate immunity (only on day 0, 1, 28 and 29)
- Training on subject diary
- Collection and review of diary

After dosing the following events will be performed **at 2 h, 4 h, 6 h, 8 h, 12 h, 24 h:**

- Vital signs (and at 0 h, before dose administration)
- Temperature (and at 0 h, before dose administration)
- (AE)/ConMed (and at 0 h, before dose administration)
- Injection site reactions & systemic events/reactions assessment

9.4.10.3.4 Ambulatory Visits

Subjects will return to the study ward for ambulatory visits on study day 3, 7 and 14 (±2), after the first vaccination and on study day 35, 42 (±2), 56 (±3), 84 (±3) after the second vaccination.
On the ambulatory visits the following events will be performed:

- Targeted Physical Examination (only on day 42)
- Vital signs/ Temperature
- 12-lead ECG (only on day 7, 14, 35, 42)
- Injection Site reaction & systemic events/ reaction assessment (only on day 3, 7, 14, 35 and 42)
- Safety Laboratory (Urine) (only on day 7, 14, 35, 42)
- Safety Laboratory (Blood) (only on day 3, 7, 14, 35, 42)
- Blood sampling for troponin (only on day 7, 14, 35, 42)
- Blood sampling for humoral immunogenicity (only on day 7, 14, 35, 42, 56, 84)
- Blood sampling for cellular response (only on day 14, 42, 56, 84)
- PBMC freezing and plasma aliquots
- Blood sampling for RNA storage (only on day 3, 7, 35)
- Blood sampling for innate immunity (only on day 3, 7, 35)
- Adverse events assessment
- Concomitant medication assessment
- Collection and review of diary

9.4.10.3.5 Ambulatory vaccination and subsequent ambulatory visits (only for subjects who gave consent to the study extension)

Subjects will return to the study ward for an ambulatory vaccination visit 12 months (±4 months) after the first immunization.

The following interventions will be performed:

- Informed Consent
- I/E Criteria check
- Update medical history
- Weight
- Physical examination
- Vital signs / Temperature
- 12-lead ECG
- Injection Site reaction & systemic events/reaction assessment
- AE/ConMed
- Alcohol Breath Test
- Urine Drug Screen
- Urine Pregnancy Test
- Safety Laboratory (Urine/Blood)
- Blood sampling for humoral immunogenicity
- Blood sampling for cellular response
- PBMC freezing and plasma aliquots
- Blood sampling for RNA storage
- Blood sampling for troponin
- Blood sampling for innate immunity
- Dosing
- Training on subject diary

After dosing the following events will be performed **at 2 h, 4 h and 6 h:**

- Vital signs (and at 0 h, before dose administration)
- Temperature (and at 0 h, before dose administration)
- (AE)/ConMed (and at 0 h, before dose administration)
- Injection site reactions & systemic events/reactions assessment

Subjects will return to the study ward for ambulatory visits on study day 1, 3, 7 and 14 (±2), after the third vaccination.
On the ambulatory visits the following events will be performed:

- Vital signs/ Temperature
- Injection Site reaction & systemic events/ reaction assessment
- Safety laboratory (urine) (only on day 7, 14)
- Safety laboratory (blood)
- Blood sampling for humoral immunogenicity (only on day 7, 14)
- Blood sampling for cellular response (only on day 7, 14)
- PBMC freezing and plasma aliquots
- Blood sampling for RNA storage (only on day 1, 3, 7)
- Blood sampling for innate immunity (only on day 1, 3, 7)

- Adverse events assessment
- Concomitant medication assessment
- Collection and review of diary

9.4.10.3.6 End of study/ Early termination visit

An end-of-study follow-up visit will be performed on day 180 (±7). The following interventions will be performed:

- Physical examination
- Vital signs/Temperature
- Weight
- AE/ConMed
- Urine pregnancy test
- Safety Laboratory (Urine and Blood)
- Blood sampling for Troponin T
- Injection site reactions & systemic events/reactions assessment
- Collection subject diary
- Blood sampling for humoral immunogenicity
- Blood sampling for cellular response
- PBMC freezing and plasma aliquots

For subjects receiving 3 vaccinations, an additional end-of-study visit will be 28 (±2 days) after the 3rd vaccination. The following interventions will be performed:

- Vital signs/Temperature
- AE/ConMed
- 12-lead ECG
- Urine pregnancy test
- Safety laboratory (urine and blood)
- Blood sampling for Troponin T
- Injection site reactions & systemic events/reactions assessment
- Collection of subject diary
- Blood sampling for humoral immunogenicity
- Blood sampling for cellular response
- PBMC freezing and plasma aliquots

9.4.10.4 Early Termination

All enrolled subjects who complete the study, discontinue early, or withdraw from the study will have the follow-up assessments and procedures completed for their safety as outlined in Table 1. Subjects who discontinue early or withdraw from the study will be replaced if they discontinue early or withdraw up to day 35.

In some cases it may be necessary for subjects to return to CTC North for additional care, confinement, and/or follow-up. Circumstances in which this may be necessary are:

- Follow-up on abnormal laboratory evaluations
- Follow-up on an ongoing AE at the final visit
- All additional safety follow-up visits will be at the discretion of the investigator and the sponsor.

9.5 Study Procedures and Examinations

Details regarding scheduled assessments and procedures to be conducted in this study are provided below. For detailed assessment of schedules refer to Table 1.

All relevant Laboratories involved in this trial work according to GLP. Continuous GLP compliance throughout the trial is assured.

9.5.1 Screening Procedures

Written, signed, and dated informed consent from the subject prior to the performance of any study related procedures must be obtained by an Investigator. Subjects will first have ample time to read the subject information before an investigator will start the information and informed consent process. The subject information/ informed consent process will be performed according to CTC North SOPs in groups of 2-5 subjects. Investigator will provide the subjects with information of the study and explain the nature of the study point by point. During this verbal subject information process subjects have already the opportunity to ask questions. After that, the subjects have the opportunity to individually ask questions in a one-to-one meeting with the Investigator. If the Investigator is convinced that the subject understands the nature and risks of the trial, and each subject had ample time for consideration and formulation of questions (which could also mean that the subjects first discuss the decision with friends or family members), and if all questions are answered the subject will asked to personally sign the informed consent form. A copy or a second original of the signed informed consent form must be given to the subjects for their records.

Screening procedures must be completed between 28 days and 2 days prior to receiving the first dose of study medication. See Table 1 for a complete list of screening procedures to be performed.

Only an authorized and trained investigator may decide on the eligibility of the subject.

9.5.1.1 Screening Failure

A screening failure is defined as a subject who has given informed consent and failed to meet at least one inclusion criteria or met at least one exclusion criteria or has not been administered IMP as defined by the protocol.

Eligible subjects who meet all inclusion and no exclusion criteria but are unable to participate in the study due to scheduling conflicts/timing will not be considered screening failures.

9.5.1.2 Re-screening of Subjects

Subjects who fail to meet all inclusion criteria or meet any exclusion criterion will not be permitted to be re-screened. Screening failures will not be enrolled into the study or receive IMP and cannot be re-screened for the study at any point.

Eligible subjects who meet all inclusion and no exclusion criteria but are unable to participate in the study due to scheduling conflicts/timing may be re-screened based on investigator's discretion and sponsor's approval should their availability to participate fall outside the screening window. In these cases, a new screening number must be assigned for each subject to be re-screened and a new informed consent form signed to confirm consent for study participation.

9.5.2 Study Examinations

Assessments are to be performed according to the schedule shown in Table 1 and Table and depend on time point of IMP administration.

9.5.2.1 Safety

Safety will be evaluated by collecting reported adverse events at regular intervals throughout the study and by the assessment of physical examination findings, vital signs, clinical laboratory parameters, ECGs, and adverse events.

9.5.2.2 Medical and Medication History

A complete medical and medication history as well as demographic information will be assessed at the time-points indicated in Table 1.

The medical history will be reviewed and recorded, including:

Medical and Medication History

- Recent ingestion of medication (30 days prior to entering the screening period)
- History of respiratory, cardiovascular, renal, gastrointestinal, hepatic, endocrine, hematological, neurological, psychiatric, musculoskeletal and other diseases.

Demographic information

- Date of Birth
- Sex

9.5.2.3 Physical Examination

A complete physical examination will be performed at the time points described in Table 1.

The physical examination will include a review of the following body systems:

- General appearance
- Skin
- Head, Eyes, Ears, Nose and Throat
- Spine/Neck/Thyroid
- Musculoskeletal
- Respiratory
- Cardiovascular
- Neurological
- Abdomen (including liver and kidneys).

Any abnormalities or changes in intensity from the screening visit noted during the review of body systems have to be documented in the medical record. Clinically significant abnormal findings discovered during a physical examination after screening will be documented either as part of medical history (subject forgot to mention an intermittent medical condition at screening) or documented as an Adverse Event or part of an Adverse Event (up from the time after dose administration), if the discovered symptom is leading to a diagnosis.

9.5.2.4 Electrocardiogram

A 12-lead ECG will be done at the time points described in Table 1. Actual ECG assessment times will be documented.

Subjects must be resting in a supine position for at least 5 minutes prior to collecting the ECG. At a minimum, the date and time of when the event was performed, the investigator's assessment and the heart rate, RR, PR, QT, and QRS intervals are to be collected. All clinically significant abnormalities will be recorded on the appropriate source documents.

9.5.2.5 Vital Signs

Blood pressure should be determined by cuff (using the same method and in the same position throughout the study). Measurements of vital signs (systolic and diastolic blood pressure as well as pulse rate) will be performed after the subject has been in a sitting position for at least 5 minutes at the time points specified in Table 1. Actual vital sign assessment times will be recorded.

All measurements of vital signs must be recorded in the appropriate source documents.

Any vital sign assessment that deviates from the scheduled assessment time set forth in the protocol will be considered a protocol deviation.

Deviations from vital signs measurements are allowed as follows:

± 30 minutes (for vital sign measurements on day 0, 1, 28 and 29 and day 0 for the 3rd vaccination)

9.5.2.6 Temperature

Temperature will be measured using a digital thermometer (orally at the time points specified in Table 1).

9.5.2.7 Height and Weight

Measurements of height and weight will be performed according to the schedule in Table 1.

Height is measured in centimeters (cm) and weight is measured in kilograms (kg). Measurements are to be taken in light clothing and socks (without shoes) with pockets emptied. The subject's height is recorded to the nearest cm and weight is recorded to the nearest 0.1 kg.

Height and weight will be measured and used to calculate the BMI using the following formula:

$$\text{BMI} = \frac{weight[kg]}{(height[m])^2}$$

9.5.2.8 Clinical Laboratory Evaluations

All laboratory assays will be performed according to the laboratory's normal procedures. Reference ranges will be supplied by the laboratory and used to assess the laboratory data for clinical significance and out-of-range pathological changes. The Investigator should assess out-of-range laboratory values for clinical significance, indicating if the value(s) are not clinically significant (NCS) or clinically significant (CS). Abnormal laboratory values that are unexpected or not explained by the subject's clinical condition may be, at the discretion of the Investigator or sponsor, repeated until confirmed, explained, or resolved as soon as possible.

The following laboratory assessments will be performed:

9.5.2.8.1 Bio-Chemistry

Blood samples (4.9 mL) for serum biochemistry will be collected into a lithium heparin separator tube at the time points described in Table 1. The following parameters will be assessed:

Sodium	Aspartate transaminase (AST)
Potassium	Alanine transaminase (ALT)
Calcium	Alkaline phosphatase (ALP)
Urea	Gamma glutamyl transferase (GGT)
Creatine kinase (total)	Total bilirubin
Albumin	Glucose
Total protein	Pancreas specific amylase
Creatinine	Lactate dehydrogenase (LDH)
Creatinine clearance (MDRD) (only at Screening)	C reactive protein (CRP)

9.5.2.8.2 Hematology

A 2.7 mL sample of blood will be drawn into a tube containing potassium ethylene diamine tetra-acetic acid **(EDTA)** anticoagulant at the time points described in Table 1. The following parameters will be assessed:

Hemoglobin	Mean corpuscular hemoglobin concentration (MCHC)
Hematocrit	White blood cell (WBC) count; total and differential
Red blood cells (RBC)	Neutrophils
Mean corpuscular volume (MCV)	Lymphocytes

	Monocytes
Platelet count	Eosinophils
Mean corpuscular hemoglobin (MCH)	Basophils
Reticulocytes	

9.5.2.8.3 Safety Urinalysis

The following parameters will be analyzed in fresh midstream urine.

pH	Ketones
Specific gravity	Bilirubin
Protein	Blood
Glucose	

Microscopic examination will be conducted if blood is detected during urinalysis. The microscopic examination will comprise of RBC, WBC, casts, and bacteria.

9.5.2.8.4 Urine Pregnancy Test

In all female participants a urine beta-HCG test will be performed on fresh midstream urine at screening and on day 180.

9.5.2.8.5 Serum Pregnancy Test

A 7.5 mL sample of blood will be drawn into a Serum Monovette and the following parameter will be assessed at the time points described in Table 1:

ß-HCG

9.5.2.8.6 Cardiac biomarker

A 4.9 mL sample of blood will be drawn into serum separator tube **(Z-Gel)** at the time points described in Table 1. The following parameters will be assessed.

Troponin T

9.5.2.8.7 Serology

During the screening period only, two blood samples of approximately 7.5 mL will be drawn into a serum separator tube **(Z- Gel)** to test for the presence of HIV, Hepatitis B and C:

HIV antibody (HIV I and HIV II)	HCV antibody screen
HBsAg	

9.5.2.8.8 Drug and Alcohol Screen

An alcohol breath test and urine screen for drugs of abuse will be performed at the time points described in Table 1. Additional drug and alcohol screens may be performed at the investigator's discretion.

Urine samples are to be tested for the following:

Methadone (MTD)	Barbiturates
Benzodiazepines	Tricyclic Antidepressants (TCA)
Cocaine	Morphine
Amphetamine/Methamphetamine (MET)	Tetrahydrocannabinol (THC)
Ecstasy	

9.5.2.8.9 Reactogenicity

To assess reactogenicity after each immunization body temperature and reaction are recorded for at least 14 days after vaccination or until any symptoms resolved if longer. According to the defined time frame of ±2 days for the ambulatory visit on day 42 recording might be shortened to day 40. During confinement at the study ward temperature and solicited injection site and systemic reactions will be assessed and recorded by a designated member of the study team. After dismissal subjects are asked to maintain a diary to record daily temperature and solicited (expected) injection site and systemic reactions for 14 days after each dosing. Subjects will be asked to maintain a second diary for another 14 days to record medicinal events. As described above, the second diary after the second immunization should be maintained at least until day 40. Subjects will be trained on the thermometer and diary use.

The following items will be recorded:

- Daily temperature (incl. the method by which it was taken)
- Daily measurement of intensity grade of all other solicited (expected) injection site and systemic reactions
- Action taken for each recorded event (none, medication taken, physician consulted, hospitalization)

9.5.2.8.10 Local Reaction Assessments

Local reactions, resulting from the IMP, will be graded using a local reaction assessment scale

Grade Description:

- 0 = None
- 1 = Mild macular or papular eruption, erythema, or induration that is asymptomatic or mildly symptomatic.
- 2 = Moderate macular or papular eruption, erythema or induration with pruritus, mild blistering or other associated moderate symptoms.
- 3 = Severe ulceration, superinfection, severe blistering, or phlebitis.
- 4 = Potentially life-threatening necrosis of the skin.

9.5.2.8.11 Adverse and Serious Adverse Events Assessments

Subjects will be questioned in a general way to ascertain if AEs have occurred (e.g. "Have you had any health problems since the last time you came to the clinic/since you were last questioned?"). This open, standardized questioning should be done discretely in order to prevent subjects from influencing each other. Spontaneous reports of AEs will also be recorded as well as AEs that are observed by the investigator or a staff member.

All AEs will be reviewed, confirmed, and classified by a qualified, designated physician.

9.5.3 Pharmacodynamic Measurements

Detailed information about blood sampling and processing of the samples are defined in the safety/lab manuals.

9.5.3.1 Immunogenicity Assays

The information on the blood volume drawn for immunogenicity assays given below refer to maximum amounts. Details on the exact blood volume are specified in the laboratory manual.

9.5.3.1.1 Enzyme-Linked Immunosorbent Assay (ELISA)

To identify binding MVA-MERS antibody responses induced by the candidate vaccine ELISA assays will be performed on serum samples (7.5 mL serum tube) drawn at the time points described in Table 1. The analysis will be performed at the Institute for Virology in Marburg, Germany according to the process defined in the Safety/ Laboratory Manual. Seroconversion will be defined as at least a two-fold rise in titers, or becoming seropositive in originally seronegative subjects.

9.5.3.1.2 Neutralization Assay

Neutralization antibody responses against MVA will be measured on the same serum sample used for ELISA (7,5 mL serum tube) obtained at the time points described in Table 1 using a luciferase based

assay in HeLa or DF-1 cells. The analysis will be performed at the Institute for Virology in Marburg, Germany according to the process defined in the laboratory manual.

9.5.3.1.3 T cell IFN-γ ELISPOT

The magnitude, kinetics and epitope targets of MVA-MERS T-cell responses will be assessed by IFN-γ ELISpot assay. The assay will be performed on cryopreserved PBMC samples (from 4 x 9 mL EDTA tubes) obtained at the time points described in Table 1. The analysis will be performed in the Virus-Immunology Research Laboratory, Heinrich-Pette-Institute, Hamburg, Germany according to the process defined in the Laboratory Manual.

Blood samples (36 mL) for T cell immunogenicity analysis (2 x 9 mL for EliSpot and 2 x 9 mL for ICS) will be drawn by direct venipuncture into four EDTA tubes, capped and mixed by inversion (x3) The actual time that the sample was obtained will be recorded on the source documents. After applying a tourniquet, venous blood will be taken with a disposable needle.

The labels will contain the following information:

Study Number

Study Day and Time point

Subject Identifier

9.5.3.1.4 Sample preservation for Intracellular Cytokine Staining for MERS-S-specific CD4 and CD8 T cell responses

The magnitude and kinetics of MERS-S-specific CD4 and CD8 T-cell responses will be determined from cryopreserved PBMC at the time points described in Table 1 using flow cytometry (from 2 x 9 mL EDTA tubes as above). The analysis will be performed by Prof. Addo’s and Dr. Schulze zur Wiesch’s group at the UKE, hosted in the Virus-Immunology Research Laboratory, Heinrich-Pette-Institute, Hamburg Germany according to the process defined in the Laboratory Manual.

9.5.3.1.5 Sample preservation for RNA analysis

In the exploratory translational research program gene expression analysis from whole blood (2.5 mL Paxgene tube) will be performed at baseline and early time points post-vaccination to investigate early innate immunity gene signatures induced by the vaccine candidate and their impact on adaptive B and T cell immunity.

9.5.3.1.6 Innate immunity

To evaluate early innate immunity gene expression signatures cryopreserved PBMC at the time points described in Table 1 will be analyzed using flow cytometry (from 2 x 9 mL EDTA tubes). Furthermore, plasma obtained after centrifugation of the EDTA tube will be analyzed via luminex multiplex assays.

9.5.3.1.7 Isolation of antigen-specific B cells

Cryopreserved PBMC will be shipped to Prof. Florian Klein at the University of Cologne for the isolation of MERS-S specific B cells using flow-cytometry.

9.5.3.2 Shipment of Immunogenicity Samples

All samples, along with the corresponding documentation, will be transported from the CTC North to the Heinrich-Pette Institute, Leibniz Institute for Experimental Virology (AG Addo), UKE campus, building N 63 for further processing.

After processing frozen serum samples for MVA-MERS antibodies will be shipped from there to the Institute of Virology in Marburg (Prof. Stephan Becker):

Institute for Virology
Philipps University Marburg
Hans Meerweinstr. 2
35043 Marburg, Germany

Phone: [redacted]
Fax:
Mobile:
Email:

After processing frozen PBMC samples for MVA-MERS B cells will be shipped from there to the laboratory for experimental Immunology (Prof. Florian Klein):

Labor für Experimentelle Immunologie
Klinik I für Innere Medizin
Zentrum für Molekulare Medizin
Universität zu Köln
Robert-Koch-Str. 21, Gebäude 66,
50931 Köln

Phone: [redacted]
Email:

9.5.3.3 Samples for cellular response and exploratory translational samples

Samples, along with the corresponding documentation, will be transported from the CTC North to:

Prof. Marylyn Addo, UKE Emerging Infections
Heinrich Pette Institute, UKE Campus, Building N 63
Martinistr. 52
20251 Hamburg, Germany

Shipment and storage procedures are defined in the Laboratory Manual.

9.5.3.4 Future Use of Stored Samples

Left over blood samples may be used in the further evaluation of an adverse event or for the subsequent evaluation of additional parameters that are identified as important to the evaluation of an individual subject or to the study, or for further research regarding the immune response to the MVA-MERS vaccine. Any unused part of the blood samples will be securely stored in Prof. Addo's laboratory for up to 15 years. Parts of the blood samples may pseudonymized be shipped to collaborators for further evaluation.

9.5.3.5 Safety Variables

Assessment of safety is performed for the safety set. Safety data include:

- Adverse events (including changes from baseline in physical examination findings)
- Clinical laboratory results
- Vital signs
- 12-lead ECGs

The safety evaluation will be based upon the review of the individual values (potentially clinically important abnormalities) and descriptive statistics (summary tables, graphics) (see section 9.8).

9.5.3.5.1 Adverse Events

The adverse events will be listed per subject using MedDRA terminology (preferred term and system organ class) and will be reported in tables summarizing the frequency of subjects with adverse events and adverse events by dosage level, by dose level and injection and by body system, the number of adverse events and number of subjects with adverse events by dosage level, by dosage level and injection, and the characteristics of adverse events.

For the hematology, clinical laboratory and the urine analysis deviations from the reference ranges will be summarized in frequency tables.

9.5.3.5.2 Clinical Laboratory

All relevant clinical variables obtained during screening, treatment phase or final examination will be reported in appropriate tables together with descriptive statistics. Clinical laboratory findings outside of the reference range will be flagged.

9.5.3.5.3 Vital Signs

For blood pressure and pulse rate descriptive statistics will be listed by sampling times (screening and follow up) according to the data reported in the source documents.

9.5.3.5.4 ECG

The results of the 12 lead ECG will be listed by sampling times (screening and follow up) according to the data reported in the source documents.

9.5.4 Pharmacogenetic Measurements

No pharmacogenetic measurements will be performed in this trial.

9.6 Data Quality Assurance

9.6.1 Quality Assurance System

Standard operating procedures are available for all activities relevant to the quality of the study. All phases of the study may be subject to audits by the quality assurance unit (QAU) of the study center. Results of these audits as well as any objections will be reported directly to the management. The QAU will issue a certificate on their activities.

9.6.2 Monitoring

An assigned and qualified clinical monitor may at any time demand information on the progress of the study by phone or in writing, or may visit the study center in order to review the original investigator folder and subject documents.

Due to the nature of the study (phase I, first in human) a monitoring width of 100% of the subject and investigator folder documents is deemed necessary to ensure subject safety and data integrity.

For this purpose a study monitor will review the investigator folder for completeness and verify the eCRF data against the source documents during frequent regular on site monitoring visits (source data verification (SDV)). The study monitor will point out any discrepancies between source data and the data captured in the eCRF. The monitor will issue electronic queries to site staff to initiate discrepancy resolution. Discrepancies which require eCRF data corrections have to be resolved by authorized site personnel by answering these monitoring queries.

Further details of the monitoring procedures will be described in a study specific monitoring plan.

9.6.3 Documentation and Data Collection

An electronic case report form (eCRF) will be used to report all clinical data required by the protocol.

Source documents (either paper or electronic files) will be used to collect and document at least the data required for transfer into the eCRF. During initial data collection, the study staff enters the information directly into these source documents. All entries on paper documents must be clearly legible and signed by the person who made the entry.

Next, site staff will transfer the study data from the source documents into the eCRF. All eCRFs will be checked for completeness and electronically signed by an investigator in order to ensure data entry accuracy.

Corrections to study documents will be dated and initialed. Reasons for the corrections will be given if deemed necessary. Corrections to eCRF entries must be electronically tracked. The date on which the correction was performed and the person will be automatically recorded by the system's audit trail.

9.6.4 Data Management

Data management will double check all eCRF entries as defined in the DMP. Quality control and data validation procedures such as programmed automatic edit and consistency checks ensure data validity and accuracy immediately at the point of entry into the clinical database. The database application which is used to capture electronic study data is fully CFR part 11 compliant. Thus, it is access restricted, demands electronic signatures, maintains an electronic audit trail and provides appropriate backup functionalities. Details of the application and eCRF configuration and all further data management procedures will be described in the data management plan (DMP).

The database will only be locked after all queries and discrepancies that may occur during data entry are resolved.

Upon request safety reports and interim analysis will be generated and provided to the respective members of the Local Safety Board.

After database lock, the data in the study data base will be exported and SAS datasets will be compiled for statistical analysis. The data will be exported in SAS transport files or other SAS-compatible format and transferred electronically to the responsible biometrician for statistical analysis. The locked SAS database will be used to generate the subject listings, tabulations, and analyses.

9.6.5 Archival of documents

CTC North will maintain the trial documents and take measures to prevent accidental or premature destruction of these documents.

All documents related to the study will be retained until at least 15 years after the end of the study. At the end of this period, CTC North will require permission from the sponsor before any documents will be destroyed or transferred.

All immunogenicity laboratory documents will be stored in the GLP archive of the University Medical Center Hamburg-Eppendorf and Philipps University Marburg for a minimum of 15 years.

The TMF will be stored in the GLP archive of the University Medical Center Hamburg-Eppendorf for a minimum of 30 years.

If documents shall be retained for a longer period, it is the responsibility of the sponsor to inform CTC North when these documents no longer need to be retained.

In case of any change concerning the archiving modalities CTC North has to inform the sponsor immediately.

9.7 Adverse Events

9.7.1 Definition of Adverse Events, Period of Observation, Recording of Adverse Events

An **Adverse Event (AE)** is any untoward medical occurrence in a clinical investigation subject administered an Investigational Medicinal Product (IMP) and that does not necessarily have a causal relationship with this treatment. An AE can therefore be any unfavorable and unintended sign (including an abnormal laboratory finding), symptom, disease or exacerbation of a pre-existing condition temporally associated with the use of a medicinal (test) product, whether or not considered related to the medicinal product (ICH Guidance E2A 1995).

All AEs, including those associated with the protocol, are collected from the time after first administration of IMP until the defined follow-up period and are to be recorded on the appropriate AE pages in the eCRF and in source documents. Where possible, a diagnosis rather than a list of symptoms should be recorded. If a diagnosis has not been made then each symptom should be listed individually.

A solicited AE is a predetermined event occurring within 14 days after application of IMP, which may reflect safety concerns related to the investigational product. The solicited AEs for this study include:

- Swelling, redness/ erythema, induration, hematoma or pain at site of injection
- Fever
- Chills
- Myalgia (described to the subject as generalized muscle aches)
- Arthralgia (described to the subject as generalized joint aches)
- Fatigue
- Headache
- Gastrointestinal symptoms (nausea, vomiting, abdominal pain, loose stool and/or diarrhea)
- Malaise

All AEs must be followed to closure (the subject's health has returned to his/her baseline status or all variables have returned to normal), an outcome is reached, stabilization (the investigator does not expect any further improvement or worsening of the event), or the event is otherwise explained regardless of whether the subject is still participating in the study. When appropriate, medical tests and examinations are performed so that resolution of event(s) can be documented.

9.7.2 Severity Categorization of AEs

The severity of AEs must be recorded during the course of the event including the start and stop dates for each change in severity. An event that changes in severity should be captured as a new event. Worsening of pre-treatment events, after initiation of investigational medicinal product, must be recorded as new AEs.

The medical assessment of severity is determined by using the following definitions:

Mild: A type of AE that is usually transient and may require only minimal treatment or therapeutic intervention. The event does not generally interfere with usual activities of daily living.

Moderate: A type of AE that is usually alleviated with additional specific therapeutic intervention. The event interferes with usual activities of daily living, causing discomfort but poses no significant or permanent risk of harm to the research subject.

Severe: A type of AE that interrupts usual activities of daily living, or significantly affects clinical status, or may require intensive therapeutic intervention.

Additionally, the severity of AEs will be graded according to the toxicity scale described in section 9.7.11. If the AE is not defined in the tables of section 9.7.11 the severity will be graded according to NCI CTCAE (current version).

The severity assessment is described more detailed in section 9.7.11.

9.7.3 Relationship Categorization

An investigator must make the assessment of relationship to IMP for each AE. The investigator should decide whether, in his or her medical judgment, there is a reasonable possibility that the event may have been caused by the IMP. If there is no valid reason for suggesting a relationship, then the AE should be classified as ‘not related’. Otherwise, if there is any valid reason, even if undetermined or untested, for suspecting a possible cause-and-effect relationship between the investigational medicinal product and the occurrence of the AE, then the AE should be considered ‘related’. The causality must be documented in the source document.

The following additional guidance may be helpful:

Term	Relationship	Definition
Related	Yes	The temporal relationship between the event and the administration of the IMP is compelling and/or follows a known or suspected response pattern to that product, and the event cannot be explained by the subject’s medical condition, other therapies, or accident.
Not Related	No	The event can be readily explained by other factors such as the subject’s underlying medical condition, concomitant therapy, or accident and no plausible temporal or biologic relationship exists between the investigational medicinal product and the event.

9.7.4 Outcome Categorization

The outcome of AEs must be recorded during the course of the study in the eCRF. Outcomes are as follows:

- Recovered/resolved
- Recovering/resolving
- Not recovered/not resolved/ ongoing
- Recovered/ resolved with sequelae
- Fatal
- Unknown

9.7.5 Clinical Laboratory Evaluations

A change in the value of a safety laboratory investigation can represent an AE if the change is clinically relevant or if, during treatment with the investigational medicinal product, a shift of a parameter is observed from a normal value to an abnormal value, or a further worsening of an already abnormal value. When evaluating such changes, the extent of deviation from the reference range, the duration until return to the reference range, either while continuing treatment or after the end of treatment with the IMP, and the range of variation of the respective parameter within its reference range, must be taken into consideration.

If, at the end of the treatment phase, there are abnormal laboratory values which were not present at baseline, further clinical or laboratory investigations should be performed until the values return to within reference range or until a plausible explanation (e.g., concomitant disease) is found for the pathological laboratory values.

The Investigator should decide, based on the above criteria and the clinical condition of a subject, whether a change in a laboratory parameter is clinically significant and therefore represents an AE.

9.7.6 Abuse, Misuse, Overdose, and Medication Error

Abuse, misuse, overdose, or medication error (as defined below) must be reported to the sponsor according to the SAE reporting procedure whether or not they result in an AE/SAE.

Abuse - Persistent or sporadic intentional intake of a study medication at a dose higher than prescribed per protocol (but below the dose defined for overdose) or when used for non-medical purpose (e.g. altering one’s state of consciousness)

Misuse - Intentional or unintentional use of a study medication other than as directed or indicated at any dose, which is at or below the dose defined for overdose. (Note: this includes a situation where the study medication is not used as directed at the dose prescribed by the protocol)

Overdose - Intentional or unintentional intake of a dose of study medication higher than the protocol prescribed dose for each subject.

Medication Error - A mistake made in prescribing, dispensing, administration, and/or use of the study medication. For studies, medication errors are reportable only as defined below.

Administration of an expired product should be considered as a reportable medication error when associated with an AE, or if otherwise appropriate.

Cases of subjects missing doses of product are not considered reportable as medication errors.

9.7.7 Serious Adverse Event (SAE) Procedures

All AEs and SAEs occurring following IMP injection and end of study visit and observed by the Investigator or reported by the subject, respectively, whether or not attributed to study intervention, will be recorded in the eCRF. All AEs that result in a subject’s withdrawal from the study will be followed up until a satisfactory resolution occurs, or until a non-study related causality is assigned (if the subject consents to this).

Besides the occurrence also the relationship an intensity of these events will be determined.

9.7.7.1 Reporting Procedures

All initial SAE reports must be reported by the Investigator to the CTC North Safety Department within 24 hours of the first awareness of the event. All SAE follow-up reports must be reported in a timely manner. The Investigator must complete, sign, and date the Serious Adverse Event Form and verify the accuracy of the information recorded on the form with the corresponding source documents (Note: source documents are not to be sent unless requested) and fax or e-mail the form to the CTC North Safety Department:

Name:	CTC North Safety
Fax number:	[redacted]
Phone number:	[redacted]
Email:	[redacted]

CTC North forwards all SAEs for review to the medical monitor of the sponsor. He makes SAEs causality and expectedness assessment and forwards it to the CTC North Safety Department. In case an SAE is

considered related to the study medication (i.e. a SUSAR occurs), the CTC North Safety Department informs the medical monitor and coordinating principle investigator (LKP). On behalf of the sponsor CTC North has to inform the competent authority and the ethics committee about the SUSAR as soon as possible but not later than 15 calendar days. SAE resulting in death, has to be reported as soon as possible but not later than 7 calendar days.

9.7.7.2 Serious Adverse Event Definition

A Serious Adverse Event (SAE) is any untoward medical occurrence (whether considered to be related to investigational medicinal product or not) that at any dose:

- Results in death
- Is life-threatening
- Requires inpatient hospitalization or prolongation of existing hospitalization
- Results in persistent or significant disability/incapacity
- Is a congenital abnormality/birth defect
- Is an Important Medical Event, i.e., an event that may jeopardize the subject and may require medical or surgical intervention to prevent one of the outcomes listed in this definition.

Hospitalizations which are the result of elective or previously scheduled surgery for pre-existing conditions which have not worsened after initiation of treatment should not be classed as SAEs. For example, an admission for a previously scheduled ventral hernia repair would not be classed as an SAE.

However, complication(s) resulting from a hospitalization for an elective or previously scheduled surgery that meets serious criteria must be reported as an SAE(s).

9.7.8 Definition of serious adverse reaction (SAR)

A **serious adverse reaction (SAR)** is an AE (expected or unexpected) that is both serious and, in the opinion of the reporting Investigator or sponsors, believed to be possibly, probably or definitely due to an IMP or any other study treatments, based on the information provided.

9.7.9 Definition of suspected unexpected serious adverse reaction (SUSAR)

A **suspected unexpected serious adverse reaction (SUSAR)** is a SAE that is unexpected and thought to be possibly, probably or definitely related to an IMP. No category of SAE has been defined as 'expected.'

9.7.9.1 SAE Onset and Resolution Dates

The onset date of the SAE is defined as the date the event meets serious criteria. The resolution date is the date the event no longer meets serious criteria, the date the symptoms resolve, or the event is considered chronic. In the case of hospitalizations, the hospital admission and discharge dates are considered the onset and resolution dates, respectively.

9.7.9.2 Fatal Outcome

Any SAE that results in the subject's death (i.e. the SAE was noted as the primary cause of death) should have fatal checked as an outcome and the resolution date of death recorded as the resolution date. For all other events ongoing at time of death that did not contribute to the subject's death, the outcome should be considered not resolved, without a resolution date recorded.

For any SAE that results in the subject's death or any ongoing events at the time of death, the action taken with the IMP should be recorded as "dose not changed" or "not applicable" (if the subject never received IMP).

9.7.10 Regulatory Agency, Independent Ethics Committee, and Investigative Site Reporting

The sponsor is responsible for SUSAR reporting to the national regulatory authorities central Independent Ethics Committees (IECs) and to inform all investigators.

9.7.11 Assessment of Severity

The severity of clinical and laboratory AE will be assessed according to the following scales.

9.7.11.1 Toxicity Grading Scale for Local Adverse Events

Table 3: Toxicity grading scale for local adverse events

Local Reaction	Grade 0<br>Normal	Grade 1<br>Mild	Grade 2<br>Moderate	Grade 3<br>Severe	Grade 4<br>Potentially life-threatening
Redness/erythema*	<25 mm	25-50 mm	51-100 mm	>100 mm	Necrosis or exfoliative dermatitis
Swelling/induration**	<25 mm	25-50 mm and does not interfere with activity	>50 mm or interferes with activity	Prevents daily activity	Necrosis
Pain	None	Does not interfere with activity	Interferes with activity	Prevents daily activity	Emergency room visit or hospitalization

*In addition to grading the measured local reaction at the greatest single diameter, the measurement should be recorded as a continuous variable.
**Induration/swelling should be evaluated and graded using the functional scale as well as the actual measurement.
From the FDA's 2007 voluntary guidance: (http://www.fda.gov/downloads/BiologicsBloodVaccines/GuidanceComplianceRegulatoryInformation/Guidances/ Vaccines/ucm091977.pdf)

9.7.11.2 Toxicity Grading Scale for Physical Observations

Table 4: Toxicity grading scale for physical observations

Observation	Grade 1	Grade 2	Grade 3	Grade 4	Grade 5
Fever (axillary)	38.0° - 39.0°C	> 39.0°C - 40.0°C	> 40.0°C for ≤ 24 hours	> 40.0°C for ≥ 24 hours	Death
Tachycardia (bpm)*	Asymptomatic, intervention not indicated	Symptomatic, non-urgent medical intervention indicated	Urgent medical intervention indicated	Life-threatening consequences, urgent intervention indicated	Death
Bradycardia (bpm)**	Asymptomatic, intervention not indicated	Symptomatic, medical intervention indicated	Severe, medically significant, urgent medical intervention indicated	Life-threatening consequences, urgent intervention indicated	Death
Hypertension**	Prehypertension (systolic BP 120-139 mmHg or diastolic BP 80-89 mmHg)	Stage 1 hypertension (systolic BP 140-159 mmHg or diastolic BP 90-99 mmHg); medical intervention indicated; recurrent or persistent (≥24 h); symptomatic increase by >20 mmHg (diastolic) or to >140/90 mmHg if previously within normal limits; monotherapy indicated	Stage 2 hypertension (systolic BP ≥160 mmHg or diastolic BP ≥100 mmHg); medical intervention indicated; more than one drug or more intensive therapy than previously used indicated	Life-threatening consequences (e.g., malignant hypertension, transient or permanent neurologic deficit, hypertensive crisis); urgent intervention indicated	Death
Hypotension	Asymptomatic, intervention not indicated	Non-urgent medical intervention indicated	Medical intervention or hospitalization indicated	Life-threatening and urgent intervention indicated	Death

Definitions

Fever	A disorder characterized by elevation of the body's temperature above the upper limit of normal.
Tachycardia	A disorder characterized by a dysrhythmia with a heart rate greater than 100 beats per minute.
Bradycardia	A disorder characterized by a dysrhythmia with a heart rate less than 60 beats per minute.
Hypertension	A disorder characterized by a pathological increase in blood pressure; a repeatedly elevation in the blood pressure exceeding 140 over 90 mmHg.
Hypotension	A disorder characterized by a blood pressure that is below the normal expected for an individual in a given environment.

*Taken after ≥10 minutes at rest; **when resting heart rate is between 60–100 beats per minute. Use clinical judgement when characterizing bradycardia among some healthy subjects, for example, conditioned athletes.

From CTCAE v4 (http://evs.nci.nih.gov/ftp1/CTCAE/CTCAE_4.03_2010-06-14_QuickReference_5x7.pdf)

9.7.11.3 Toxicity grading scale for systemic AEs excluding the physical observations listed above

Table 5: Toxicity grading scale for systemic AEs

Systemic sign/symptom	Grade 1<br>Mild	Grade 2<br>Moderate	Grade 3<br>Severe	Grade 4<br>Potentially life-threatening
Headache	No interference with activity	Some interference with activity	Significant; prevents daily activity	Medical consultation and/or hospitalization
Fatigue	No interference with activity	Some interference with activity	Significant; prevents daily activity	Medical consultation or hospitalization
Myalgia	No interference with activity	Some interference with activity	Significant; prevents daily activity	Medical consultation and/or hospitalization
Arthralgia	No interference with activity	Some interference with activity	Significant; prevents daily activity	Medical consultation and/or hospitalization
Chills	No interference with activity	Some interference with activity	Significant; prevents daily activity	Medical consultation and/or hospitalization
Sweats	No interference with activity	Some interference with activity	Significant; prevents daily activity	Medical consultation and/or hospitalization
Subjective Fever	No interference with activity	Some interference with activity	Significant; prevents daily activity	Medical consultation and/or hospitalization
Nausea	No interference with activity	Some interference with activity not requiring medical intervention	Prevents daily activity and requires medical intervention	Medical consultation and/or hospitalization
Vomiting	No interference with activity	Some interference with activity not requiring medical intervention	Prevents daily activity and requires medical intervention	Medical consultation and/or hospitalization
Abdominal Pain	No interference with activity	Some interference with activity not requiring medical intervention	Prevents daily activity and requires medical intervention	Medical consultation and/or hospitalization
Diarrhea	No interference with activity	Some interference with activity not requiring medical intervention	Prevents daily activity and requires medical intervention	Medical consultation and/or hospitalization
Other systemic symptoms	No interference with activity	Some interference with activity	Prevents daily activity	Medical consultation and/or hospitalization

From the FDA's 2007 voluntary guidance: (http://www.fda.gov/downloads/BiologicsBloodVaccines/GuidanceComplianceRegulatoryInformation/Guidances/ Vaccines/ucm091977.pdf)

9.8 Statistical Methods Planned in the Protocol and Determination of Sample Size

9.8.1 Statistical and Analytical Plan

Details for the statistical evaluation of the results will be given in a separate statistical analysis plan (SAP).

9.8.1.1 Software to be used

All statistical calculations will be carried out using SAS language and procedures (SAS 9.2 or higher version SAS-Institute, Cary NC, USA).

9.8.1.2 Eligibility for Statistical Evaluation

Eligibility of subjects will be determined within the data review meeting (DRM). The following patient populations will be investigated:

- Safety population: All subjects receiving at least once the study medication will be included into the safety evaluation (safety collective).
- Per protocol population: All subjects who have received both injections and who have not withdrawn within the first 7 days after the second injection will be included for the per protocol analysis.

All safety endpoints will be investigated in the safety population. As a sensitivity analyses, the analysis of the safety endpoints will be repeated in the per protocol population. All other endpoints will be investigated in the per protocol population only.

9.8.1.3 Statistical analyses

For metric variables, the arithmetic mean (Mean), the standard deviation (SD), coefficient of variation (CV), absolute minimum (Min) and maximum (Max) and median (Med) will be reported per dose level. For parameters that appear to be log-normally distributed, the geometric mean (GeoM) and the coefficient of variation (CV) may be reported.

Laboratory values and their change from baseline will be summarized and displayed graphically by dose level. Values outside of the reference range will be flagged and tabulated. Unless otherwise specified, baseline is defined as the time-point closest to but prior to the first administration of the vaccine.

Categorical variables will be summarized by dose level in frequency tables (number and percentages).

The individual subject values will be listed.

It is not planned to test any hypotheses in a confirmatory sense. If deemed necessary, 95% confidence intervals will be provided for means (metric variables) and percentages (categorical variables).

Interim analyses may be performed upon request of the sponsor.

9.8.2 Determination of Sample Size

For this FIH study no formal criteria are available in order to fix the sample size. The MVA vaccine is licensed and has been tested in various clinical trials as listed in table 15 of the IB [13]. 24 subjects (10 subjects on dose level 10^7 pfu, 10 subjects on dose level 10^8 pfu and 4 subjects on placebo) were enrolled in a dose escalation study of MVA-BN [16]. In a phase I/IIa study of MVA-H5-sfMR with 80 subjects, 20 subjects received a dose of 10^7 pfu (10 subjects in one immunization, 10 subjects in two immunizations) and 20 subjects received a dose of 10^8 pfu (10 subjects in one immunization, 10 subjects in two immunizations) [17]. Therefore, N=24 subjects (12 subjects per dose level) are considered sufficient to identify major safety risk and to prove the concept.

9.9 Changes in the conduct of the study or planned analysis

Modifications of the protocol are permitted only if they are authorized by the sponsor and the investigator in writing.

Deviations and changes to the study protocol will be classified by the sponsor and the study center as:

<u>Note-to-File</u>: This refers to clarifications which are not considered changes of the protocol.

<u>Study protocol amendment</u>: This refers to changes of the protocol. If they fulfill the criteria as set out in according to §10 (1) GCP-V they need to be approved by the IEC or the competent authority or both of

it. Changes to the study protocol may also induce revision of the subject information sheet/informed consent form. Accordingly, subjects undergoing trial assessment procedures at the time of implementation of the change have to be given the amended version and have to be asked for consent to continue on this amended trial.

9.10 Local Safety Board

During the trial, the Local Safety Board will review and judge the safety, tolerability, after each dose-step and prior to the next dose-step of the trial. Based on these data the Local Safety Board will/may decide

- Whether there will be a next dose cohort and if the planned dose for the next dose cohort has to be reduced.
- Whether there will be added additional dose step(s) or dose step(s) will be repeated.

Evaluation will take the results of the pre-clinical studies and the ongoing phase I studies in prior mentioned other countries into account.

An interim safety report, summarizing the safety (AEs, vital signs, 12-lead ECGs, and laboratory safety data) will be prepared and reviewed by the Principal Investigator and Medical Monitor before the second immunization for each dose cohort.

Planned dose escalation will occur after the second immunization of all subjects of the 1st dose group following a review of the interim safety report summarizing the safety (AEs, vital signs, 12-lead ECGs, and laboratory safety data) and any available immunological data by the Principal Investigator and Medical Monitor.

The dose for the next escalation step may be reduced if there are concerns about the safety. A dose level may be repeated and furthermore, additional dose step(s) may be added but not exceeding dose of $1x10^8$ pfu MVA-MERS. The decision to repeat and/or to modify the dose level and/or to add additional dose steps will be taken by the Local Safety Board.

In addition, a Local Safety Board meeting will be conducted whenever safety relevant data occur that might have an influence on the trial.

Members of the Local Safety Board will be the medical monitor, as well as the Principal Investigator, or at least one of the deputies and a representative of the sponsor.

At a minimum the holding rules (criteria for termination of the study) specified in the protocol will automatically apply (if activated) for not proceeding with a higher dose.

The decisions of the Local Safety Board meeting will be briefly summarized by CTC North and signed by all members involved in the meeting and the decision. The summarized safety report of each Local Safety Board Meeting will be submitted to the competent national authority (Paul-Ehrlich-Institute) and the independent ethics committee (Ethik-Kommission der Ärztekammer Hamburg) for notification.

10 REPORTS

All reports to the sponsor will be in English. The sponsor will receive the original final report. A copy of the report will be archived at the archives contracted by the study center.

The final report is the property of the sponsor. Publication of the report or of part of it may only be allowed when authorized by the sponsor in consultation with the study center.

10.1 Final Report

All clinical, analytical and statistical results will be presented in a final report. The outline of this report will accord to the EFPIA/CPMP document "Structure and Content of Clinical Study Reports" of July 17, 1996.

The sponsor will receive an electronic copy of the eCRF for archival.

10.2 Additional reports

Upon completion of the study, a short report will be sent to the ethics committee, stating any undesired event and indicating whether study objectives have been attained. Short reports to the authorities after study termination will be provided as required by law

11 REFERENCES

1 Zaki AM, van Boheemen S, Bestebroer TM, Osterhaus AD, Fouchier RA. Isolation of a novel coronavirus from a man with pneumonia in Saudi Arabia. N Engl J Med. 2012 Nov 8; 367(19): 1814-20.

2 http://www.who.int/emergencies/mers-cov/en/

3 Memish ZA, Zumla AI, Al-Hakeem RF, Al-Rabeeah AA, Stephens GM. Family cluster of Middle East respiratory syndrome coronavirus infections. N Engl J Med. 2013 Jun 27;368(26):2487-94.

4 van Boheemen S, de Graaf M, Lauber C, Bestebroer TM, Raj VS, Zaki AM, Osterhaus AD, Haagmans BL, Gorbalenya AE, Snijder EJ, Fouchier RA. Genomic characterization of a newly discovered coronavirus associated with acute respiratory distress syndrome in humans. MBio. 2012 Nov 20; 3(6). pii: e00473-12.

5 Raj VS, Smits SL, Provacia LB, van den Brand JM, Wiersma L, Ouwendijk WJ, Bestebroer TM, Spronken MI, van Amerongen G, Rottier PJ, Fouchier RA, Bosch BJ, Osterhaus AD, Haagmans BL. Adenosine deaminase acts as a natural antagonist for dipeptidyl peptidase 4-mediated entry of the Middle East respiratory syndrome coronavirus. J Virol. 2014 Feb;88(3):1834-8.

6 Costello DA, Millet JK, Hsia CY, Whittaker GR, Daniel S. Single particle assay of coronavirus membrane fusion with proteinaceous receptor-embedded supported bilayers. Biomaterials. 2013 Oct;34(32):7895-904.

7 Chan PK, Chan MC. Tracing the SARS-coronavirus. J Thorac Dis. 2013 Aug;5(Suppl 2):S118-21.

8 Du L, Zhao G, Kou Z, Ma C, Sun S, Poon VK, Lu L, Wang L, Debnath AK, Zheng BJ, Zhou Y, Jiang S. Identification of a receptor-binding domain in the S protein of the novel human coronavirus Middle East respiratory syndrome coronavirus as an essential target for vaccine development. J Virol. 2013 Sep; 87(17):9939-42.

9 Agnihothram S, Gopal R, Yount BL Jr, Donaldson EF, Menachery VD, Graham RL, Scobey TD, Gralinski LE, Denison MR, Zambon M, Baric RS. Evaluation of serologic and antigenic relationships between middle eastern respiratory syndrome coronavirus and other coronaviruses to develop vaccine platforms for the rapid response to emerging coronaviruses. J Infect Dis. 2014 Apr;209(7):995-1006.

10 Zhao G, Du L, Ma C, Li Y, Li L, Poon VK, Wang L, Yu F, Zheng BJ, Jiang S, Zhou Y. A safe and convenient pseudovirus-based inhibition assay to detect neutralizing antibodies and screen for viral entry inhibitors against the novel human coronavirus MERS-CoV. Virol J. 2013 Aug 26;10:266.

11 Song F, Fux R, Provacia LB, Volz A, Eickmann M, Becker S, Osterhaus AD, Haagmans BL, Sutter G. Middle East respiratory syndrome coronavirus spike protein delivered by modified vaccinia virus Ankara efficiently induces virus-neutralizing antibodies. J Virol. 2013 Nov;87(21):11950-4.

12 Sutter G, Moss B. Nonreplicating vaccinia vector efficiently expresses recombinant genes. Proc Natl Acad Sci U S A. 1992 Nov 15;89(22):10847-51.

13 Investigator's Brochure MVA-MERS-S Version 2.0, 29.09.2017

14 http://www.fda.gov/downloads/BiologicsBloodVaccines/GuidanceComplianceRegulatoryInformation/Guidances/ Vaccines/ucm091977.pdf

15 http://evs.nci.nih.gov/ftp1/CTCAE/CTCAE_4.03_2010-06-14_QuickReference_5x7.pdf

16 Walsh SR., Wilck MB, Dominguez DJ, Zablowsky E, Bajimaya S, Gagne LS, Verrill KA, Kleinjan JA, Patel A, Zhang Y, Hill H, Acharyya A, Fisher DC, Antin JH, Seaman MS, Dolin R, Baden LR. Safety and immunogenicity of modified vaccinia Ankara in hematopoietic stem cell transplant recipients: a randomized, controlled trial. J Infect Dis. 2013 Jun 15; 207(12).

17 Kreijtz JH, Goeijenbier M, Moesker FM, van den Dries L, Goeijenbier S, De Gruyter HL, Lehmann MH, Mutsert Gd, van de Vijver DA, Volz A, Fouchier RA, van Gorp EC, Rimmelzwaan GF, Sutter G, Osterhaus AD. Safety and immunogenicity of a modified-vaccinia-virus-Ankara-based influenza A H5N1 vaccine: a randomised, double-blind phase 1/2a clinical trial. Lancet Infect Dis. 2014 Dec;14(12).
