# Supplementary material for: Increased neutralization and IgG epitope identification after MVA-MERS-S booster vaccination against Middle East respiratory syndrome
Source: Nat Commun. 2022 Jul 19;13:4182. doi: 10.1038/s41467-022-31557-0 (PMC9295877; doi:10.1038/s41467-022-31557-0)
Supplement: Supplementary file 3 — Description of Additional Supplementary Files [file 41467_2022_31557_MOESM3_ESM.pdf]

Description of Additional Supplementary Files

File Name: Supplementary Data 1
Description: Table S3: EUROIMMUN ELISA OD values.

File Name: Supplementary Data 2
Description: IgM and IgA microarray.
