# Supplementary material for: Increased neutralization and IgG epitope identification after MVA-MERS-S booster vaccination against Middle East respiratory syndrome
Source: Nat Commun. 2022 Jul 19;13:4182. doi: 10.1038/s41467-022-31557-0 (PMC9295877; doi:10.1038/s41467-022-31557-0)
Supplement: Supplementary file 6 — Reporting Summary [file 41467_2022_31557_MOESM6_ESM.pdf]

nature portfolio

Corresponding author(s): Marylyn M. Addo

Last updated by author(s): Jun 1, 2022

Reporting Summary

Nature Portfolio wishes to improve the reproducibility of the work that we publish. This form provides structure for consistency and transparency in reporting. For further information on Nature Portfolio policies, see our Editorial Policies and the Editorial Policy Checklist.

Statistics

For all statistical analyses, confirm that the following items are present in the figure legend, table legend, main text, or Methods section.

n/a	Confirmed	
☐	☒	The exact sample size (*n*) for each experimental group/condition, given as a discrete number and unit of measurement
☐	☒	A statement on whether measurements were taken from distinct samples or whether the same sample was measured repeatedly
☐	☒	The statistical test(s) used AND whether they are one- or two-sided<br>*Only common tests should be described solely by name; describe more complex techniques in the Methods section.*
☒	☐	A description of all covariates tested
☐	☒	A description of any assumptions or corrections, such as tests of normality and adjustment for multiple comparisons
☐	☒	A full description of the statistical parameters including central tendency (e.g. means) or other basic estimates (e.g. regression coefficient) AND variation (e.g. standard deviation) or associated estimates of uncertainty (e.g. confidence intervals)
☐	☒	For null hypothesis testing, the test statistic (e.g. *F*, *t*, *r*) with confidence intervals, effect sizes, degrees of freedom and *P* value noted<br>*Give P values as exact values whenever suitable.*
☒	☐	For Bayesian analysis, information on the choice of priors and Markov chain Monte Carlo settings
☒	☐	For hierarchical and complex designs, identification of the appropriate level for tests and full reporting of outcomes
☐	☒	Estimates of effect sizes (e.g. Cohen's *d*, Pearson's *r*), indicating how they were calculated

Our web collection on statistics for biologists contains articles on many of the points above.

Software and code

Policy information about availability of computer code

Data collection	Electronic case report forms (eCRF): secuTrial® database version 5.1.0.20. Microsoft Excel®, multiple versions (latest: version 16.58), Genepix 4000B fluorescence scanner (Molecular Devices, San José, CA, USA), Odyssey Scanner (LI-COR Biotechnology Inc., Lincoln, NE, USA) Tecan Infinite F200 (Tecan, Männedorf, Switzerland), Behring ELISA Prozessor III system (Behring, Marburg, Germany), ImmunoSpot® Image analyzer (CTL Europe GmbH, Bonn, Germany), Olympus microscope BX60 (Olympus, Hamburg, Germany).
Data analysis	GraphPad Prism version 8.4.2, PepSlide Analyzer software (version 1.5.8, SICASYS Software GmbH, Heidelberg, Germany), python 3 (version 3.7.1) with numpy, pandas, re (regular expression), csv, and openpyxl packages.

For manuscripts utilizing custom algorithms or software that are central to the research but not yet described in published literature, software must be made available to editors and reviewers. We strongly encourage code deposition in a community repository (e.g. GitHub). See the Nature Portfolio guidelines for submitting code & software for further information.

Data

Policy information about availability of data

All manuscripts must include a data availability statement. This statement should provide the following information, where applicable:

- Accession codes, unique identifiers, or web links for publicly available datasets
- A description of any restrictions on data availability
- For clinical datasets or third party data, please ensure that the statement adheres to our policy

The data used in this study are provided in the Supplementary Information or Source Data file as deidentified participant data. The study protocol is available in the Supplementary Information of this manuscript. Source data are provided with this paper.

Field-specific reporting

Please select the one below that is the best fit for your research. If you are not sure, read the appropriate sections before making your selection.

- [x] Life sciences
- [ ] Behavioural & social sciences
- [ ] Ecological, evolutionary & environmental sciences

For a reference copy of the document with all sections, see nature.com/documents/nr-reporting-summary-flat.pdf

Life sciences study design

All studies must disclose on these points even when the disclosure is negative.

Sample size	For the first-in-human study of MVA-MERS-S, which builds the base for the work presented here, no formal criteria were available in order to fix the sample size. Based on ample experience with the MVA vaccine and on sample sizes usually used in Phase 1 trials, a sample size of n=24 was deemed sufficient for a proof-of-concept study and for safety assessments (see also chapter 9.8.2 of the study protocol, "determination of sample size"). All individuals who completed the study were then offered a booster vaccination and n=10 individuals accepted. They were all included in the work presented here.
Data exclusions	No data was excluded.
Replication	Safety reporting was monitored. Binding and neutralizing antibody levels were measured by two distinct assays, respectively, and the individual assays showed a strong inter-assay correlation. For each binding antibody assay (enzyme-linked immunosorbent assay (ELISA)) samples were measured in replicates of two. For the virus neutralization test (VNT) four replicates were used, while one replicate was used for the plaque-reduction neutralization test (PRNT), which correlated with the VNT. In the microarray assay and immune fluorescence assay (IFA), one replicate was used. The Middle East respiratory syndrome coronavirus (MERS-CoV) IFA and MERS-CoV neutralization test also showed a strong correlation, cross-validating the assays (see Fig. S3 of the manuscript). For all assays that used replicates (ELISAs and VNT), all attempts at replication were successful.
Randomization	In the original study assessing a primary vaccination regimen of MVA-MERS-S, participants were successively assigned to treatment arms and no randomization was performed (see chapter 9.4.4 of the study protocol). As a booster vaccination, all individuals received the same treatment. Since this trial represents a proof-of-concept study, only descriptive statistics were used and covariates were not controlled for.
Blinding	Since this study represented a Phase 1 trial, no blinding was performed. In the original trial, individuals were successively allocated to escalating open-label dose levels to ensure their safety (see chapter 9.4.7 of the study protocol). As a booster vaccination, all individuals received the same treatment.

Reporting for specific materials, systems and methods

We require information from authors about some types of materials, experimental systems and methods used in many studies. Here, indicate whether each material, system or method listed is relevant to your study. If you are not sure if a list item applies to your research, read the appropriate section before selecting a response.

Materials & experimental systems

n/a	Involved in the study	
[ ]	[x]	Antibodies
[ ]	[x]	Eukaryotic cell lines
[x]	[ ]	Palaeontology and archaeology
[x]	[ ]	Animals and other organisms
[ ]	[x]	Human research participants
[ ]	[x]	Clinical data
[x]	[ ]	Dual use research of concern

Methods

n/a	Involved in the study	
[x]	[ ]	ChIP-seq
[x]	[ ]	Flow cytometry
[x]	[ ]	MRI-based neuroimaging

Antibodies

Antibodies used	Goat anti-human IgG-Fc fragment, (cross-adsorbed) DyLight 680, 0.5 mg/ml, Bethyl Laboratories, Montgomery, TX, USA, catalog number A80-304D6, polyclonal, lot number A80-304D6-6; goat anti-human IgM (mu chain) DyLight 549, 1.0 mg/ml, Rockland Immunochemicals Inc., Limerick, PA, USA, catalog number 609-142-007, polyclonal, lot number 22634; mouse anti-MERS-CoV-nucleocapsid, SinoBiological, Eschborn, Germany, monoclonal, catalog number 40068-MM10, clone ID 10; rabbit anti-human IgG-HRP, 1.3 g/L, Dako, Santa Clara, CA, USA, catalog number P0214, polyclonal, lot number 20084897; goat anti-human IgA (alpha chain) antibody DyLight 800, 1.0 mg/ml, Rockland Immunochemicals Inc., Limerick, PA, USA, catalog number 609-145-006, polyclonal, lot number 32937; AlexaFluor488-labeled goat anti-human IgG, 1 mg/ml, Jackson, Baltimore, PA, USA, polyclonal, catalog number 109-545-088, affinity purified IgG (H+L), RRID AB_2337838; human anti-MERS-CoV-S antibody, 100 µg/ml, Detai Bio-Tech Co., Nanjing, China, clone m336, lot number P061119.
Validation	All secondary antibodies were acquired commercially and were validated by the manufacturer.

Eukaryotic cell lines

Policy information about cell lines

Cell line source(s)	Human hepatocellular carcinoma cell, Huh-7, JCRB0403, JCRB cell bank of Okayama University. Vero B4 cells DSMZ No. ACC33.
Authentication	The cell lines were not further authenticated after purchase.
Mycoplasma contamination	We confirm that all cell lines tested negative for mycoplasma contamination.
Commonly misidentified lines (See ICLAC register)	None.

Human research participants

Policy information about studies involving human research participants

Population characteristics	As this was a Phase 1 clinical trial, only healthy and young (age 18-55) men and women were eligible to participate in the clinical trial. Since the trial was conducted at a single center in Hamburg, Germany, the study population was not ethnically diverse. Of the ten participants who received a booster vaccination, all participants were Caucasian, female, young (age 18-40) and healthy (see Table S1).
Recruitment	The participants of the original trial were recruited through public advertisement (see Koch et al., LID 2020). For the study presented here, all of the participants from the original trial were contacted via phone call and informed about the possibility to participate in an extension of the original study to receive an additional booster vaccination. Since the study was a Phase 1 single-center trial (as explained above) and only individuals from the original trial were able to participate in the study extension, the participants were rather homogeneous (healthy, Caucasian females aged 18-40 years). The results are therefore not representative for a more heterogeneous group and have to be validated in larger clinical trials.
Ethics oversight	The study protocol was reviewed and approved by the Competent National Authority (Paul-Ehrlich-Institute) and the Ethics Committee of the Hamburg Medical Association (reference number PVN5531).

Note that full information on the approval of the study protocol must also be provided in the manuscript.

Clinical data

Policy information about clinical studies

All manuscripts should comply with the ICMJEguidelines for publication of clinical research and a completedCONSORT checklist must be included with all submissions.

Clinical trial registration	ClinicalTrials.gov Identifier NCT03615911
Study protocol	The full trial protocol can be found in the Supplementary Information provided with the manuscript.
Data collection	This study was a open-label single-center trial conducted in Hamburg, Germany. The booster vaccinations were administered from March 13th to March 18th, 2019, and individuals were followed-up for 28 days after the booster vaccination (last participant last visit April 15th, 2019). The initial trial assessing primary immunization of MVA-MERS-S (including recruitment and a 180-day observation period) had been conducted between Dec 17, 2017, and June 5, 2018. All data collection was performed in Hamburg, Germany. The clinical research organization Clinical Trial Center North in Hamburg performed the operative and regulatory project management of the trial; the University Medical Center Hamburg-Eppendorf was the Sponsor or this investigator-initiated trial.
Outcomes	The primary objective was to assess the overall tolerability and safety of the MVA-MERS-S vaccine. Local and systemic reactogenicity were measured for 14 days following the booster vaccination using a diary, and adverse events were assessed at all study visits (B:D0, B:D1, B:D3, B:D7, B:D14, B:D28). Changes in safety laboratory measures were also assessed at all study visits. Serious adverse events were assessed throughout the study period (End-of-Study on B:D28). The secondary objective was to evaluate MVA-MERS-S-specific antibody responses after MVA-MERS-S vaccination, which were assessed by measuring MERS-CoV-S-specific antibody levels via ELISA. An exploratory objective was the detailed characterization of vaccine-induced humoral responses.
